# Dissection of an Anaerobic Fungal Cellulosomal Endoglucanase: Impact of the Dockerin Module on Activity, Thermostability, and Flexibility

**DOI:** 10.1021/acsomega.5c00685

**Published:** 2025-05-14

**Authors:** Viviane Brito Andrade, Robson Tramontina, Dnane Vieira Almeida, Geizecler Tomazetto, Viviam M. da Silva, Frank Gabel, Yolanda M.B. Marcello, Ana Ligia Scott, Fabio Marcio Squina, Wanius Garcia

**Affiliations:** a Centro de Ciências Naturais e Humanas (CCNH), Universidade Federal do ABC (UFABC), Avenida dos Estados 5001, Bairro Bangu, Santo Andre, SP CEP 09280-560, Brazil; b Department of Biochemistry and Tissue Biology, Institute of Biology, 28132 Universidade Estadual de Campinas (UNICAMP), Rua Monteiro Lobato 255, Campinas, SP CEP 13083-970, Brazil; c School of Medicine, Department of Pediatrics, 12317 University of Pittsburgh, 4401 Penn Avenue, Pittsburgh, Pennsylvania 15224-1334, United States; d Institut de Biologie Structurale (IBS), CEA, CNRS, UGA, 71 Avenue des Martyrs, CS 10090,Grenoble 38000, France; e Centro de Matemática, Computação e Cognição (CMCC), Universidade Federal do ABC (UFABC), Avenida dos Estados 5001, Bairro Bangu, Santo Andre, SP CEP 09280-560, Brazil; f Laboratório de Ciências Moleculares, Universidade de Sorocaba (UNISO), Rodovia Raposo Tavares, km 92,5, Sorocaba, SP CEP 18023-000, Brazil

## Abstract

Cellulosomal cellulases possess an extra noncatalytic module denominated dockerin, which interacts with the scaffolding via cohesion modules to organize the enzymes within the cellulosome. Given the lack of previously solved experimental atomic structures for modular anaerobic fungal cellulases containing dockerin modules, here we employed structural modeling, molecular dynamics simulations, small-angle X-ray scattering, and biochemical analyses to gain new insights into the structure and function of cellulosomal endoglucanase from the anaerobic gut fungus Piromyces finnis (*Pf*GH5). Our results revealed that *Pf*GH5 has a nonglobular conformation in solution, exhibiting high molecular flexibility characterized by two principal collective motions: bending and twisting. The removal of the dockerin module decreased the thermostability of the catalytic domain. Interestingly, the removal of the dockerin module resulted in a slight increase in the optimal temperature and pH values of the catalytic domain and favored the random attack on soluble cello-oligosaccharides. The absence of the carbohydrate-binding module led to a slightly reduced activity of the catalytic domain on less soluble substrates. Taken together, our findings indicate that the dockerin module influences both the thermostability and the activity of the catalytic domain. Moreover, the high flexibility in the region encompassing the dockerin module most likely plays an important role in enzyme function. This study provides a valuable basis for further investigation of the role of the dockerin modules in anaerobic fungal cellulases.

## Graphical abstract

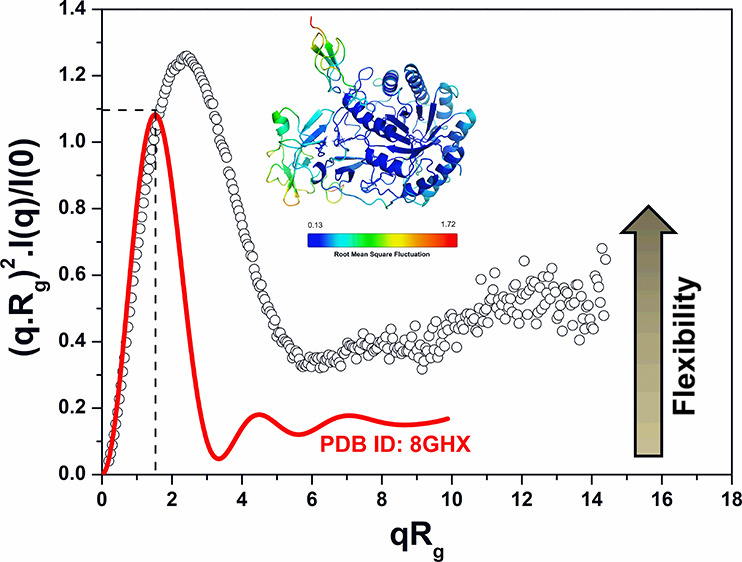

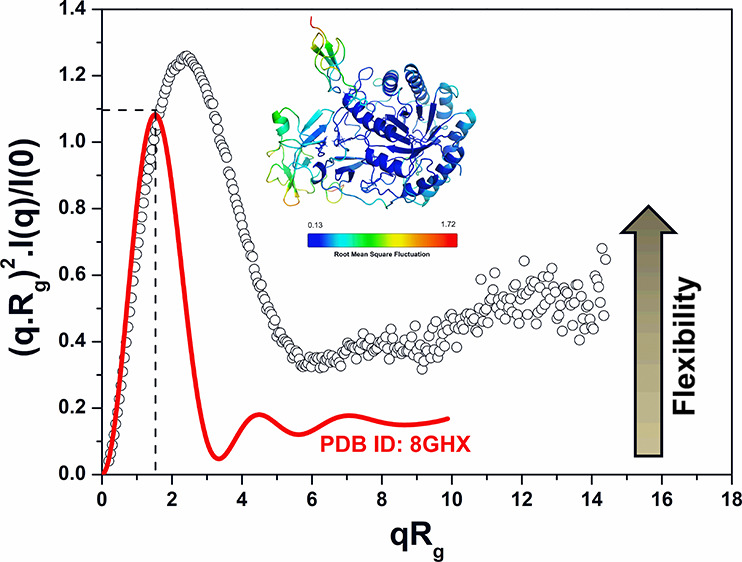

## 1 Introduction

Cellulose, a natural and renewable biopolymer with vast availability, represents an attractive feedstock for bioethanol and other bioproduct production.^1,2^ Cellulases are enzymes that catalyze the breakdown of cellulose to produce oligosaccharides, cellobiose, and glucose. These enzymes are classified into three main groups: endoglucanases (EC 3.2.1.4), cellobiohydrolases (EC 3.2.1.91), and β-glucosidases (EC 3.2.1.21).^3–6^ The classical model of cellulose hydrolysis to glucose relies on the synergistic action of these three cellulase groups.^7^ Endoglucanases cleave the amorphous regions of cellulose chains into shorter fragments, while cellobiohydrolases liberate cellobiose from the ends of these chains. Finally, β-glucosidases complete the breakdown by converting cellobiose into glucose.^3,8^ Beyond the synergy among the three cellulase groups, enzymatic hydrolysis of cellulose is further enhanced by lytic polysaccharide monooxygenases (LPMOs). These are oxidative metalloenzymes that cleave and oxidize cellulose.^9–11^

Several bacteria and fungi are classified as cellulolytic microorganisms due to their ability to secrete cellulases and degrade cellulose.^12–16^ Fungi are recognized as the most efficient cellulose degraders in nature. They possess the capability to produce and secrete a large amount of cellulases utilizing low-cost substrates.^7,16–18^ In contrast to aerobic fungi, which have been extensively studied for their genetic, enzymatic, and biotechnological potential (e.g., *Trichoderma* and *Aspergillus*), anaerobic fungi are less studied.^16,19,20^

Aerobic and anaerobic microorganisms employ distinct strategies for cellulose degradation. Aerobic microorganisms secrete different cellulases typically containing two key domains: a catalytic domain responsible for breaking down cellulose and a noncatalytic carbohydrate-binding module (CBM) that enhances the efficiency of the catalytic module when they are attached.^21^ In contrast, anaerobic microorganisms secrete cellulases containing an extra noncatalytic module named dockerins. These dockerin modules interact with the noncatalytic scaffolding containing numerous cohesion modules. This interaction organizes the enzymes into a highly structured extracellular multienzymatic complex known as cellulosome.^22–24^

Cellulases, derived from both fungal and bacterial sources, often exhibit a modular architecture. This modular design allows them to possess multiple structural and functional units, potentially contributing to their efficiency in cellulose breakdown.^25^ Surprisingly, despite their prevalence and importance, an experimental atomic structure of a modular anaerobic fungal cellulase containing a dockerin module has yet to be elucidated. This lack of structural knowledge also hinders our understanding of how dockerin modules influence the stability and activity of the catalytic domains within cellulosomes.

To address these uncertainties regarding the influence of dockerin modules and to gain insights into the structure and function of anaerobic fungal cellulases, we employed a combined approach of structural modeling, molecular dynamics simulations, small-angle X-ray scattering, and biochemical analyses. We focused on modular endoglucanase from the anaerobic gut fungus Piromyces finnis (*Pf*GH5). These studies aim to elucidate the impact of the dockerin module on the activity, thermostability, and structural flexibility of the enzyme. We further investigate how the dockerin module affects the enzyme’s specific activity profile and its hydrolytic degradation pattern.

## 2 Materials and Methods

### 2.1 Molecular Modeling Using AphaFold2 Program and Refining

We predicted a model for the endoglucanase from the anaerobic fungus P. finnis employing the Alphafold2 program,^26^ with six recyclings, in which this protein sequence contains 519 amino acids. Specifically, the FASTA sequence of *Pf*GH5 was provided to retrieve similar sequences from databases, such as UniRef90. AlphaFold2 then assessed the presence or absence of tertiary or quaternary structures for the target molecule in the Protein Data Bank (PDB) and computed a distance matrix among these sequences for co-evolutionary calculations. Finally, the deep learning module Evoformer was applied. The five best models, before and after a rapid minimization using the Amber99sb force field^27^ (performed by the software itself), were obtained. The Predicted Local Distance Difference Test (pLDDT) scores were obtained with an average of 97 and 60 for the structure. After analyzing the structures using the software Procheck,^28,29^ we select the best minimization model. We performed 100,000 steps of energy minimization followed by a 10 ns equilibration with a 2 fs timestep using the NAnoscale Molecular Dynamics (NAMD) 2.14 software at a temperature of 310 K. The particle mesh Ewald (PME) method was used to treat electrostatic interactions^30^ using the all-atom CHARMM36 force field.^31–33^ Additionally, a 100 ns production run was conducted in NAMD for models to improve the model validation. In the MD simulation, we employed Langevin dynamics and used the Nose–Hoover Langevin piston to control the target pressure set to 1 atm, with the period of the damping coefficient of 1 ps and the Langevin piston and decay parameters set to 100 and 50 fs, respectively.

### 2.2 Protocol Overview: Calculation of Normal Modes (NM)

Macromolecules adopt several conformations in solution, depending on their structure and shape, which determine their dynamics and function. These conformations exhibit unique structure-encoded dynamic properties that profoundly influence their biological function.^34,35^ Several studies have indicated that these dynamic properties are primarily determined by the topology of native contacts.^35^ In recent years, studies have established the role of structural dynamics, also called intrinsic dynamics, facilitating, if not driving, the interactions and functioning of biomolecular systems in the cell.^36^ Therefore, it is crucial to explore different conformations to model dynamical processes and determine stable states.^37^ The flexibility of a protein is directly correlated to its function, influencing its interactions with other molecules.

We applied the same protocol described in Gasparini et al. (2023):^38^ minimization of the model generated by the AlphaFold program. Initially, harmonic constraints were applied and progressively decreased (250–0 kcal mol^–1^ Å^–2^), and for each constraint value, 500 steps of gradient conjugate (CG) were applied. After the CG minimization, the constraints were removed, and 2 × 10^5^ steps of adopted basis Newton Raphson (ABNR) were performed using the program CHARMM. In the last step, the normal modes were calculated using the facility of CHARMM called DIMB (Iterative Mixed-Basis Diagonalization), considering all the protein atoms and with the force field CHARMM36.^31,39^ We analyzed the flexibility assessed by root mean square fluctuation (RMSF) and the collective motions of the endoglucanase to identify the kind of collective motions.

### 2.3 Sampling the Conformations Using Hybrid Methods

After calculating the normal modes, we computed the root mean square fluctuations (RMSFs) of the carbons for each mode. Based on the obtained values, we selected the modes exhibiting greater flexibility (7 to 20) for sampling along the normal modes. The first hybrid method we applied, VMOD, is a facility implemented in the CHARMM program that combines Normal Modes Analysis and free Molecular Dynamics to sample the protein’s conformational space. The displacements along the modes were achieved by using a series of low-temperature MD simulations followed by energy minimizations, which allowed a complete free movement of the protein, including the side chains. The structures were displaced from −3.0 to +3.0 Å (mass weighted root mean square (MRMS) values) along the selected normal modes using steps of 0.2 Å that generated 31 structures for each one of the first 20 normal modes with low frequency. The procedure was similar to that used by Batista et al. (2011) and Gasparini et al. (2023).^38,40^ A force constant of the harmonic restraining potential (*K*_d_) was increased to ensure a slow convergence toward the average mode-based displacement. The restraining force *K*_d_ value was increased from 1000 to 10,000 kcal mol^–1^ Å^–2^ during successive 10 ps MD simulations. Velocities were randomly assigned corresponding to a temperature of 30 K in the MD simulations. A final MD simulation was achieved with a *K*_d_ value of 20,000 kcal mol^–1^Å^–2^ followed by an additional 1000 steepest descents. This procedure generated 620 conformers from the initial model obtained with the AlphaFold2 program.

### 2.4 Molecular Dynamics with Excited Normal Modes

Another hybrid method we employed to sample the conformational heterogeneity for endoglucanases is called MDeNM. It is a multireplica method to investigate conformational heterogeneity. It was designed to enhance conformational exploration in a subspace defined by low-frequency normal modes, considering the couplings with the localized motions occurring within the Cartesian space.^41^ Each replica starts from an equilibrated initial structure whose normal modes are calculated, and the system is driven dynamically along a direction obtained by a linear combination of selected lowest-frequency modes. To deal with the complexity of the conformational space and the vast number of possible normal mode combinations we utilized the program CHARMM.^34,35,42^ Twenty replicas were generated, each with 20 excitations, where the excitation temperature of the system was 7.5 K. Next, we performed a principal component analysis (PCA) using the MDeNM structures to identify conformational distributions along the first three PCA components, and subsequently, we conducted a clustering process using the k-means method. These methodologies were carried out using the MDAnalysis library in Python.^43,44^

### 2.5 Recombinant Expression and Purification

Solomon et al. (2016) characterized the transcriptional profile of *Piromyces* in response to cellulosic substrates, including filter paper, Avicel, and reed canary grass.^45^ This previous study identified in *Piromyces* cultures on lignocellulose upregulated transcripts containing Carbohydrate-Active enZYme (CAZYme) domains, connected to noncatalytic dockerin domains, which are expected to play a role in extracellular cellulosome self-assembly.^45^ The upregulated transcript comp11848_c2_seq3 was depicted in response to Avicel from this transcriptomic data set, encoding a glycoside hydrolase from family 5, a carbohydrate-binding module (CBM type 1), and putative dockerin domains. The corresponding coding region of comp11848_c2_seq3 had the signal peptide and C-terminal regions trimmed and codon optimized for E. coli prior to being synthesized by GenScript.

The full-length protein was designated as *Pf*GH5 (amino acid residues from 1 to 519, disregarding the signal peptide, Table S1), and the isolated GH5 catalytic domain was designated as *Pf*GH5_cat (amino acid residues from 1 to 318). The expression and purification of recombinant *Pf*GH5 and *Pf*GH5_cat were carried out as described previously.^12^ Briefly, the recombinant products were expressed in E. coli BL21­(DE3) in soluble form and purified by a two-step protocol employing both affinity and size-exclusion chromatography.^12^ Their purities were checked by sodium dodecyl sulfate-polyacrylamide gel electrophoresis (SDS-PAGE). The concentration of each recombinant product was determined by the absorbance at 280 nm and using a theoretical extinction coefficient based on the amino acid composition obtained from the ProtParam utility available on the Expasy server.^46^

### 2.6 Small-Angle X-ray Scattering (SAXS)

SAXS measurements were collected at the beamline BM29 BioSAXS at the European Synchrotron Radiation Facility (ESRF) in Grenoble, France, using an X-ray wavelength of 0.99 Å in a quartz capillary of 1 mm. The *Pf*GH5 was measured at 20 °C and different protein concentrations (3, 6, and 12 mg/mL) in 100 mM sodium phosphate buffer adjusted at pH 6 using the batch mode. The sample-to-detector distance was set as 2.81 m. Buffer scattering was recorded before and after each sample scattering. The X-ray patterns were recorded using a two-dimensional Pilatus 1M detector (by Dectris). The integration of SAXS patterns was performed with the FIT2D (www.esrf.eu/computing/scientific/FIT2D). The radii of gyration (*R*_g_) were determined by the Guinier approximation and using the GNOM program.^47^ The distance distribution functions, *P*(*r*), and maximum dimensions (*D*_max_) were evaluated with the GNOM program.

### 2.7 Circular Dichroism (CD) Spectroscopy

The thermal unfolding of *Pf*GH5 and *Pf*GH5_cat was monitored by CD spectroscopy using a Jasco J-815 spectropolarimeter coupled to a Peltier control system. CD spectra were measured from samples (5 μM in 100 mM sodium phosphate buffer adjusted at pH 6) in a 1 mm path length quartz cuvette and were the average of eight accumulations within the range of 190 to 260 nm using a scanning speed of 100 nm/min, a spectral bandwidth of 1 nm, and a response time of 0.5 s. For thermal unfolding characterization, ellipticity changes at 220 nm were monitored as the temperature was increased from 20 to 80 °C at a rate of 5 °C/min followed by a 3 min equilibration period at each temperature. The irreversible two-state nature of the thermal unfolding process was assumed for the analysis of the ellipticity data. The fraction of denatured protein, *U*_F_, was calculated from the relationship *U*_F_ = (θ_n_ – θ_obs_)/(θ_n_ – θ_d_) and *U*_N_ + *U*_F_ = 1, in which θ_obs_ is the ellipticity of the sample at a particular condition and θ_d_ and θ_n_ are the values of ellipticity characteristic of the denatured and native states.

### 2.8 Enzymatic Assays

The optimal pH and temperature of the *Pf*GH5 and *Pf*GH5_cat were carried out by measuring the amount of reducing sugar released from oat β-glucan (β-1,3 and β-1,4-glucans) using the 3,5-dinitrosalicylic acid (DNS) method.^48^ The enzymatic conditions were carried out in buffers ranging from pH 3.5 to 8.5 and in the temperature range from 20 to 80 °C. To conduct the pH variation tests in the range of 3.5 to 8.5, the following buffers were used, all at a concentration of 100 mM: Tris-HCl (pH 7.5–8.5), sodium phosphate (pH 5.0–7.0), and sodium acetate (pH 3.5–4.5). All reactions for pH and temperature tests were performed by mixing 5 μL of *Pf*GH5 or *Pf*GH5_cat (final concentration 0.6 μM), 45 μL of 100 mM buffer, and 50 μL of the oat β-glucan substrate (0.5%, w/v) and then incubating for 30 min.^12^ The reactions were stopped by adding 100 μL of DNS, and the mixture was then immediately boiled for 5 min at 100 °C. The color intensities were measured in a double-beam UV–visible spectrophotometer (Biachrom Libra) at 540 nm. The calibration curves were constructed by using glucose as a standard. One unit of enzymatic activity corresponds to the amount of enzyme required to release 1 μmol of reducing sugar per minute. All enzymatic activity assays were carried out in triplicate. To evaluate the influence of carbohydrate-binding module on enzymatic activity against different substrates, a panel was constructed for *Pf*GH5 and *Pf*GH5_cat using four substrates at a concentration of 0.5% (w/v): carboxymethylcellulose (CMC, β-1,4-glucan), barley β-glucan (β-1,3- and β-1,4-glucan), β-d-mannan (β-1,4-mannan), and oat β-glucan (β-1,3- and β-1,4-glucan). The reactions were carried out under the previously determined optimal conditions for 30 min.

### 2.9 Analysis of the Hydrolytic Cleavage Pattern

The analysis of the hydrolytic cleavage pattern of *Pf*GH5 and *Pf*GH5_cat was carried out according to the protocol previously described.^12^ Cellohexaose (C6) from Megazyme was used as a substrate. Each reaction had a final volume of 100 μL, and aliquots were taken after 2, 4, 8, 16, 40, and 60 min; the reactions were stopped at high temperature (100 °C) for 10 min. Samples (1 μL injections) were analyzed by ion exchange chromatography (HPAEC) coupled with pulsed amperometry (PAD) in the ICS-6000 Dionex (Thermo Scientific). The column employed was a CarboPac PA1, 2 × 250 mm, set at 30 °C with a running time of 30 min and a flow rate of 0.25 mL/min. The sugars were eluted isocratically in a mobile phase of 30 mM NaOH. The data were analyzed by using the Chromeleon Chromatography Data System.

## 3 Results and Discussion

### 3.1 The Endoglucanase from P. finnis Has a Modular Architecture

No prior studies have elucidated an experimental atomic structure of an anaerobic fungal cellulase containing a dockerin module.^49^ Despite efforts to crystallize *Pf*GH5, we were unable to obtain suitable crystals for protein structure determination. Therefore, we employed the AlphaFold2 program to predict the tertiary structure of *Pf*GH5, providing valuable insights into its three-dimensional architecture. The final three-dimensional model predicted by AlphaFold2 for modular *Pf*GH5 is shown in Figure 1, with the structure colored according to the predicted Local Distance Difference Test (pLDDT) score (Figure S1). The Ramachandran plot of the final model (Figure S2) demonstrates excellent quality, with more than 96% of residues occupying the most favored regions. A large portion of the structure exhibits a high pLDDT value (pLDDT > 90), particularly in the GH5 domain (residues 3–316) and the CBM10 domain (residues 418–454), whereas the CBM1 (residues 481–519) and CBM10 domains displayed lower values (pLDDT < 70). A similar trend was observed for the predicted aligned error (PAE) values, which are provided in the Supporting Information (Figure S3). Following equilibration, we believe that a slight structural improvement occurred, as the α-carbon contact map after equilibration (Figure S4) appears to be more diffuse compared to the corresponding map of the AlphaFold2-generated structure (Figure S4A). The Supporting Information also includes data on the secondary structure evolution over time, analyzed using DSSP (Dictionary of Secondary Structure in Proteins), as well as the average protein contact map of α-carbons for Classical Molecular Dynamics (Figures S5 and S6).

**Figure 1:**
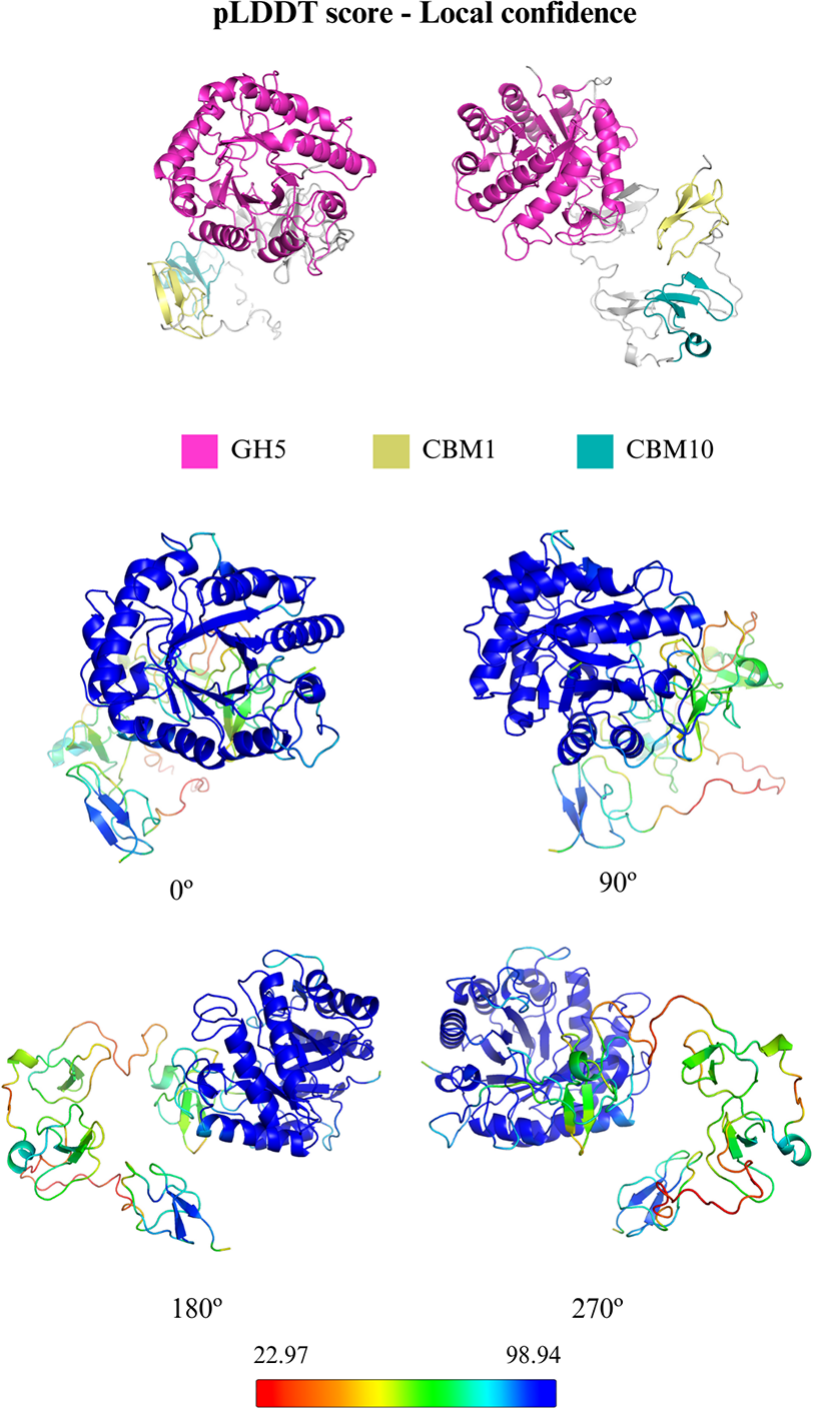
Modular architecture of the *Pf*GH5 model built by AlphaFold2 colored by pLDDT. The final predicted model is shown above, while four different views of the model are shown below. The N-terminal catalytic domain (GH5, residues from 3 to 316) is connected to the C-terminal carbohydrate-binding module family 1 (CBM1, residues from 481 to 519) through the double dockerin module family 10 (CBM10, residues from 418 to 454).

### 3.2 The AlphaFold2 Model Was Minimized and Equilibrated with a Classical Molecular Dynamics

A DSSP plot, showing the secondary structure changes along the Classical Molecular Dynamics, has been added to the Supporting Information (Figure S5). A plot of the radius of gyration (*R*_g_) for the Classical Molecular Dynamics (100 ns) is presented in Figure S7. In the next step, we performed an MDeNM simulation, as discussed in the methodology, allowing us to sample a significant variety of conformations relative to the minimized structure. Using this ensemble of structures, we conducted a principal component analysis (PCA) considering the first three principal components (PCs) (Figure 2).^50^

**Figure 2:**
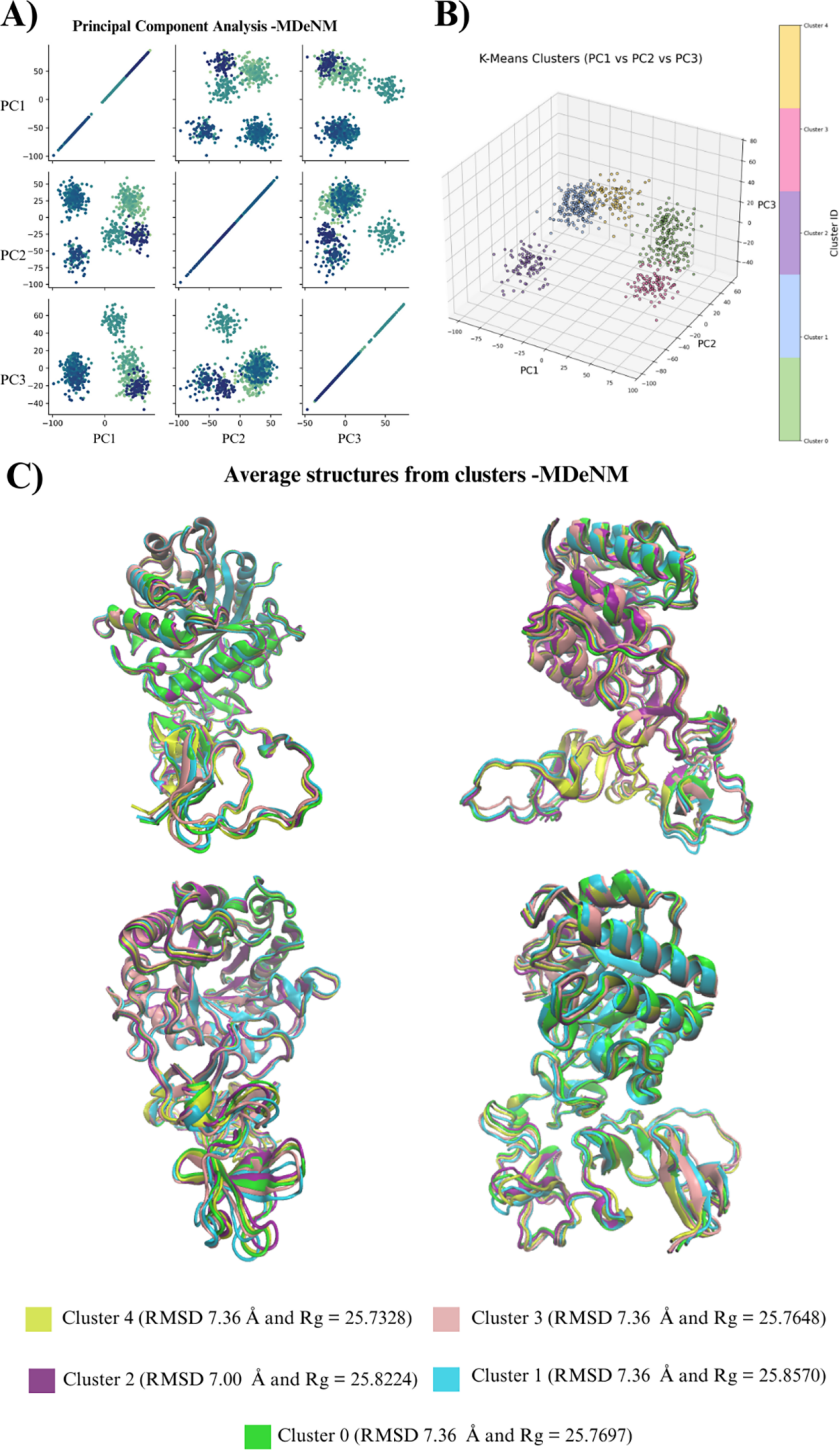
Clustering analysis of the MDeNM structures. (A) Principal component analysis (PCA) for the first three orthogonal PCs. (B) The k-mean-clustered structures from MDeNM. (C) Representative structures for each cluster.

We obtained well-separated regions in the PCA space, indicating the presence of significant conformational differences (Figure 2A). Subsequently, using k-means clustering via PCA,^50^ we identified five well-defined clusters (0 to 4), which can be ranked in terms of population size from most to least populated as follows: 4 > 1 > 0 > 2 > 3 (Figure 2B). Additionally, clusters 0, 1, 3, and 4 exhibit similar RMSD values relative to the minimized structure, whereas cluster 2 presents a slightly lower value. On the other hand, the average radii of gyration were very similar between each cluster and slightly lower than for classical MD. Figure 2C displays the average structures of these clusters, representing the atomic mean positions of the structures within each grouping, and they average RMSD and *R*_g_. The *R*_g_ values were calculated with Visual Molecular Dynamics (VMD).^51^ Figure S8 presents the RMSD histogram for the conformations obtained by VMOD and RMSD calculated between the conformations generated by MDeNM and the initial model, and in Figure S9, we have the RMSD of the protein structure over the simulation time.

The final structures predicted by MNeNM consist of a GH5 domain connected to CBM1 through CBM10.^12^ The GH5 catalytic domain adopts the (β/α)_8_-barrel fold structure common to many GH5 enzymes.^52,53^ The dockerin domains in anaerobic fungi have been described as belonging to the CBM10 family and may be responsible for the assembly of a cellulase multiprotein complex, similar to the cellulosome found in certain anaerobic bacteria.^54^ The dockerin structure comprises two tandem identical dockerin modules, each formed by a three-stranded β-sheet and a short α-helix.^55,56^ This fold is consistent with the double dockerin structure previously reported for the cellulase Cel45A from Piromyces equi.^57^ The CBM1 is a small and compact module, which adopts a characteristic two-stranded β-sheet fold common to the family 1 CBMs.^58–60^

Moreover, the percentage of secondary structure contents of the *Pf*GH5 domains (GH5, CBM1, and CBM10) were calculated using the DSSP (Database of Secondary Structure Assignments) algorithm, comparing experimental data with our *in silico* models. X-ray crystallography data were used for the GH5 domain of Piromyces finnis (PDB code: 8GHX), while NMR (nuclear magnetic resonance) data were employed for CBM1 and CBM10 of Trichoderma reesei (PDB code: 7YHI) and Piromyces equi (PDB code: 2J4N), respectively. The results, presented in Table S2, demonstrate a strong agreement between the *in silico* predictions and experimental data, with α-helix and β-strand ratios falling within the expected range values.^49,61^

### 3.3 The Endoglucanase from P. finnis Presents Two Types of Collective Motions

We calculated 29 normal modes (NM) with low frequency and observed two types of collective motions that can be important for *Pf*GH5: bending and twist (Figure 3).

**Figure 3:**
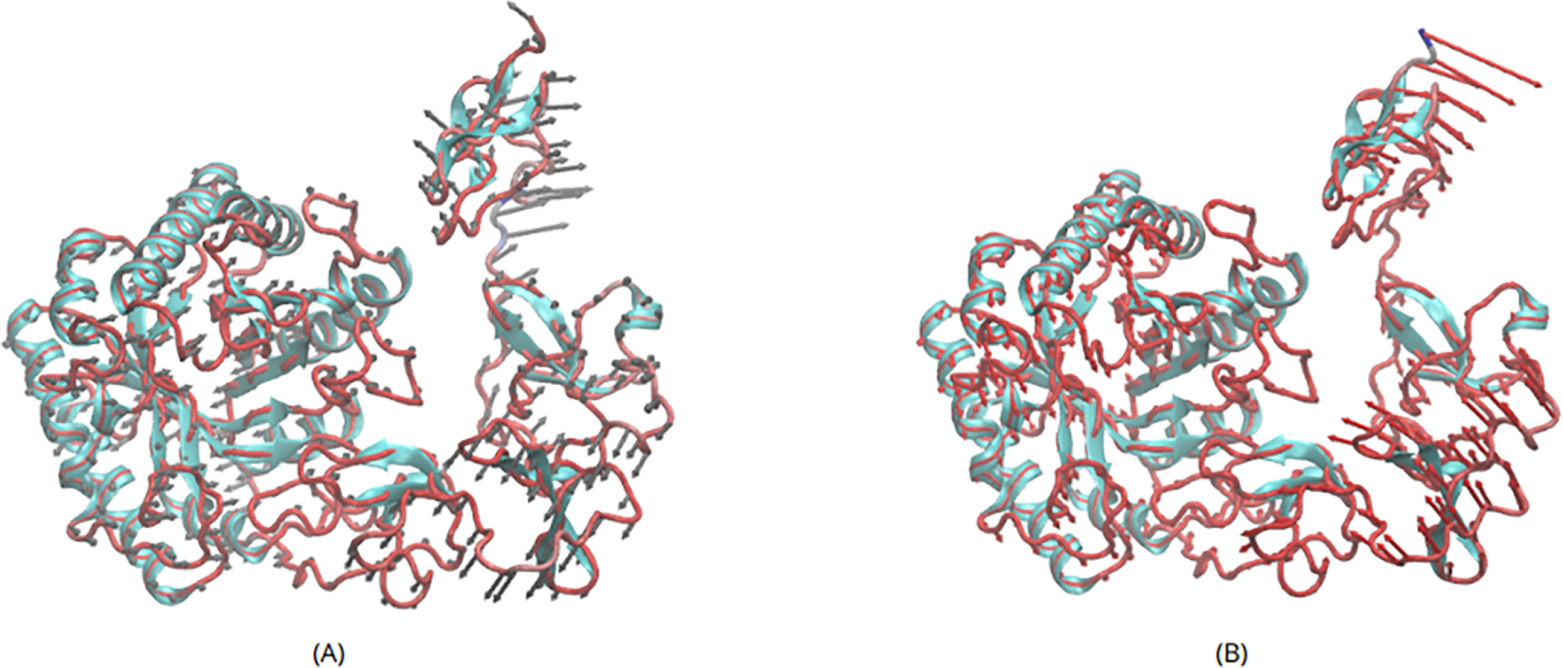
General view of flexibility and collective motions. (A) Twist motion evolves in the region, encompassing residues from 329 to 519, corresponding to normal low-frequency modes. (B) Bending motion that increases and decreases the distance between the β-sheet (involving the Cys505 and Ile506) and the loop composed of residues Asn446, Gly447, and Glu448. This motion allows the modular structure of *Pf*GH5 to be unpacked and packed.

Analysis of both the α-carbon (C_α_) fluctuation plot for all proteins (Figure S10) and the model colored by root mean square fluctuation (RMSF) (Figure 4) reveals increased flexibility in the region encompassing residues from 329 to 519 (CBM10 and CBM1 modules). These two motions, bending and twisting, and the flexibility observed in the region encompassing residues from 329 to 519 likely play an important role in the enzyme function. These collective motions and flexibility in *Pf*GH5 induce conformational changes (unpacked and packed) that may contribute to the enzyme’s ability to accommodate and release diverse cellulose substrates from the catalytic domain.

**Figure 4:**
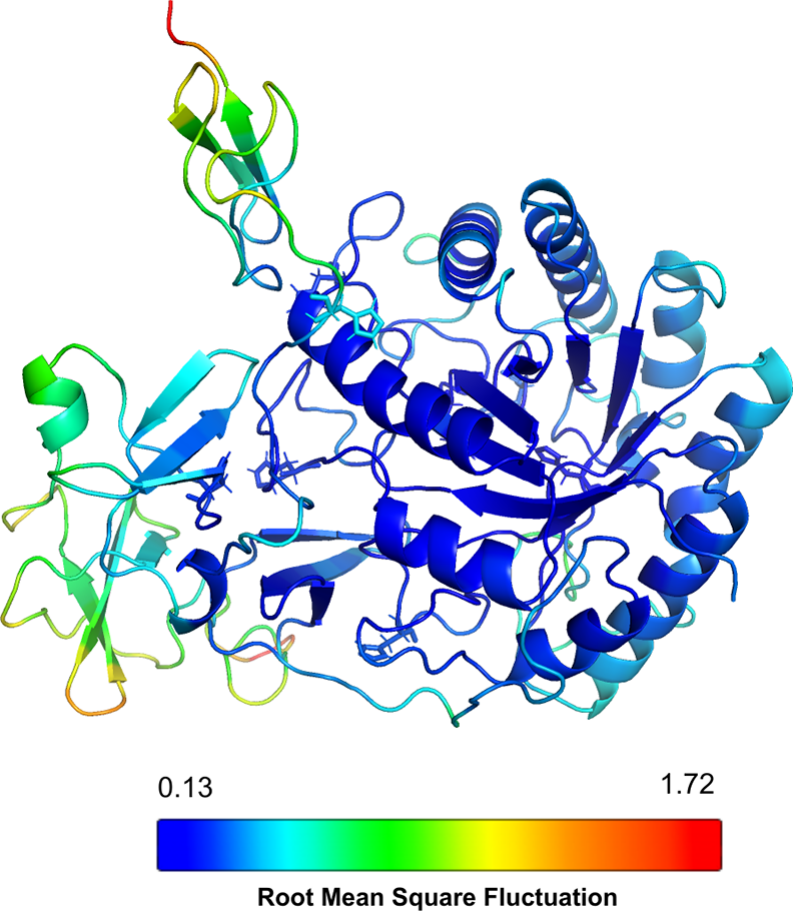
The model of *Pf*GH5 is colored by the root mean square fluctuation (RMSF) from the normal modes analysis. The region encompasses residues from 329 to 519 (CBM10 and CBM1 modules) demonstrated to be more flexible.

Following Normal Modes Analysis (NMA), we employed the VMOD hybrid method to investigate the relationship between the conformational variability depicted by low-frequency normal modes and the range of compact to extended conformations observed in *Pf*GH5. This method generated 620 distinct structures, revealing fluctuations in the distances between the GH5 catalytic domain and the CBM10 module compared to the initial model. To delve deeper into the conformational diversity, we employed a second hybrid method, MDeNM, which generated an additional 700 conformations. Compared to VMOD, this approach offered a more comprehensive exploration of conformational heterogeneity. As shown in Figure S8, the histograms of RMSD for all generated conformations reveal a broader diversity of structures for MDeNM compared with VMOD. This wider range of RMSD values underscores the high flexibility of the protein.

### 3.4 The Endoglucanase from P. finnis Has a Nonglobular Shape with High Flexibility in Solution

SAXS provides valuable information about the overall shape, size, and flexibility of a protein in solution. Therefore, to gain experimental insights into the structure of *Pf*GH5, we submitted the full-length enzyme to SAXS analysis.^62^ *Pf*GH5 was successfully expressed and purified as described previously (Figure S11).^12^ The X-ray scattering curve, measured at a concentration of 12 mg/mL, is shown in Figure 5.

**Figure 5:**
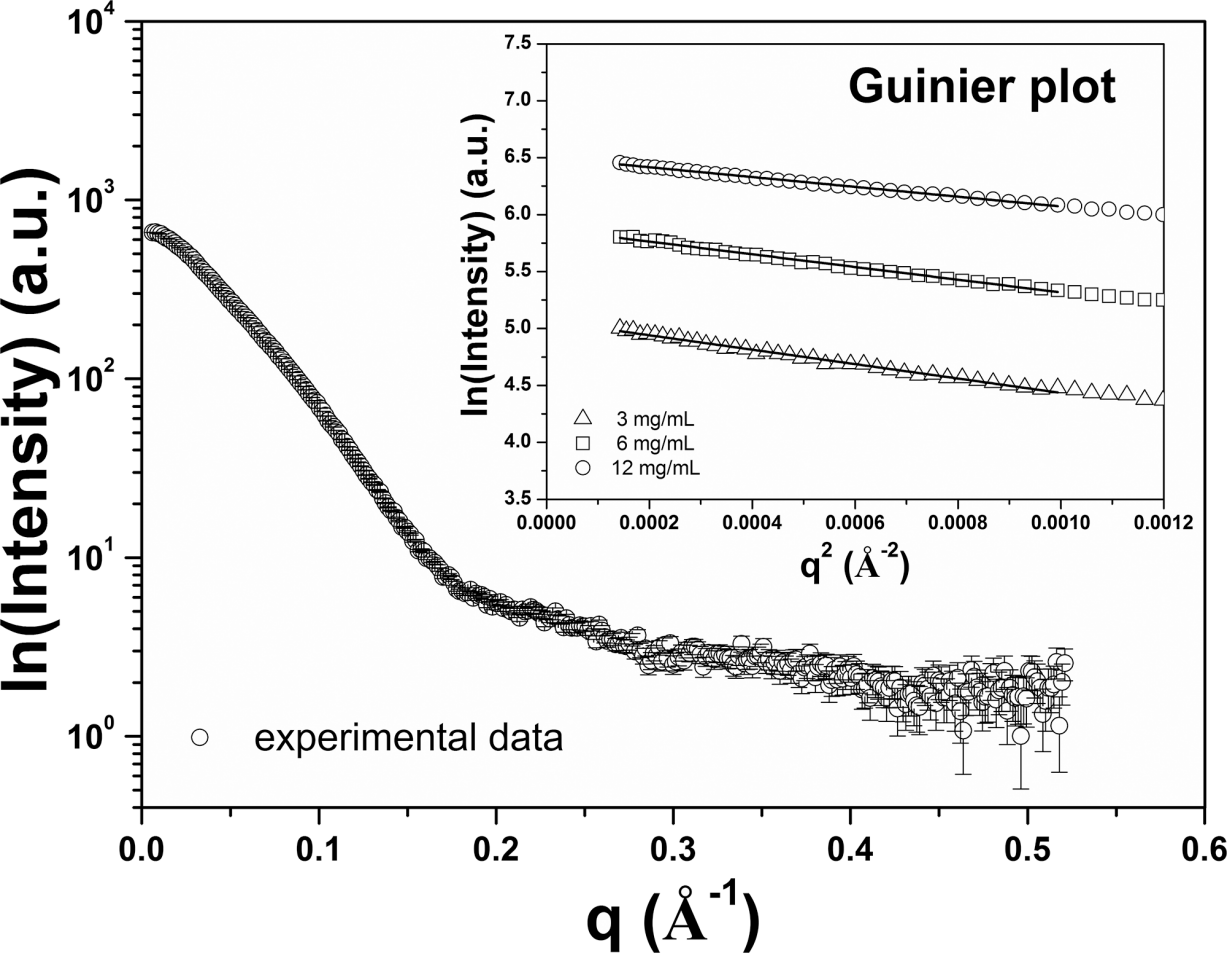
Experimental SAXS curve was measured for full-length *Pf*GH5 at 12 mg/mL and 20 °C (black circles with error bars). Inset: Guinier plots were at 3, 6, and 12 mg/mL.

The radii of gyration (*R*_g_) calculated using Guinier approximation (inset of Figure 5, *R*_g_.*q*_max_ < 1.0) at 3, 6, and 12 mg/mL were 43 ± 1, 40 ± 1, and 35 ± 1 Å, respectively. The linearity of the Guinier plots suggests good monodispersity of the samples. However, the decrease in *R*_g_ with increasing protein concentration indicates the presence of interparticle interactions. Also, SAXS is a powerful tool for characterizing the protein flexibility in solution. To gain deeper insights into the structural flexibility of *Pf*GH5 in its native state, we analyzed the experimental X-ray scattering curve using the dimensionless Kratky plot (Figure 6).

**Figure 6:**
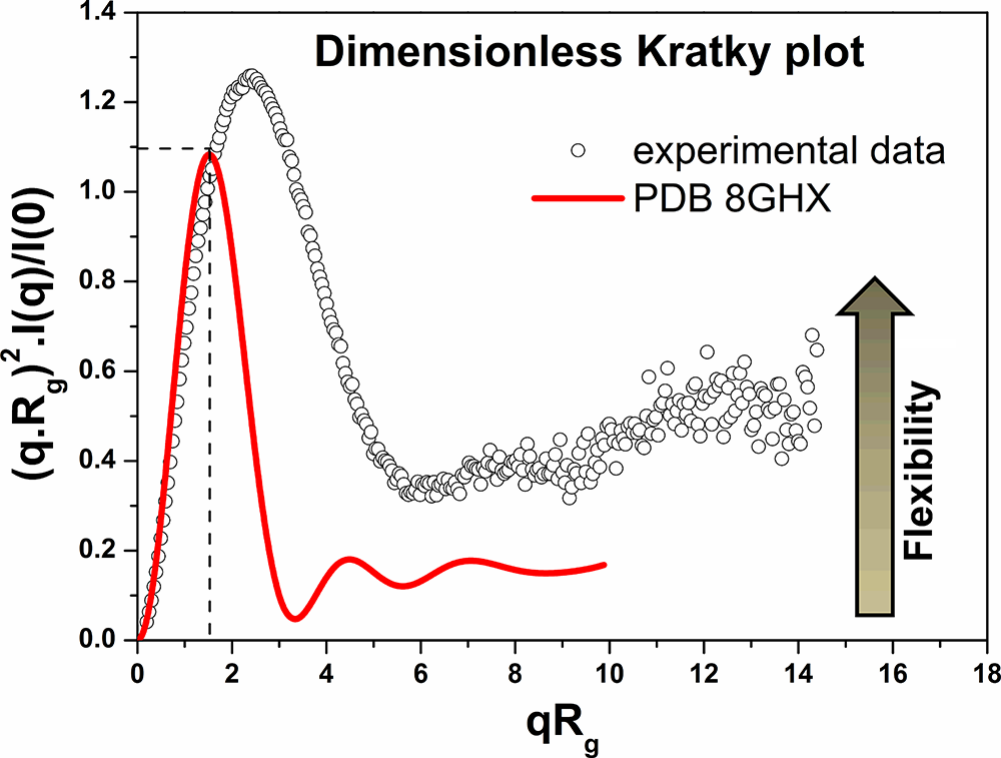
Dimensionless Kratky plot generated from the SAXS curve for *Pf*GH5 (black circles) and the theoretical SAXS curve of the CelD GH5 catalytic domain from P. finnis (PDB ID: 8GHX).

We compared the theoretical X-ray scattering curve calculated for the CelD GH5 catalytic domain from P. finnis (PDB ID: 8GHX) to the experimental X-ray scattering curve measured for *Pf*GH5. The theoretical curve for CelD GH5 (Figure 6, red line) exhibits a well-defined peak with maximum near 1.1 for *q*.*R*_g_ = $\sqrt{3}$ , decaying to near zero. As expected, this is consistent with a nearly globular tightly packed protein with limited flexibility in solution. In contrast, the experimental curve for *Pf*GH5 (Figure 6, black circles) exhibits a more pronounced peak with maximum exceeding 1.1 and shifted to higher *q*.*R*_g_ (*q*.*R*_g_ > $\sqrt{3}$ ), indicating a nonglobular structure. Furthermore, the elevated baseline that plateaus instead of decaying to zero throughout the curve (Figure 6) reflects the high structural flexibility of *Pf*GH5 in solution. The observed high flexibility, likely conferred by the dockerin module, might grant the catalytic domain greater freedom of movement, potentially enhancing its enzymatic function within the native cellulosome. The high flexibility of a cellulosome-bound cellulase enables it to reach hidden cellulose chains within the substrate and facilitates its movement and interaction with other enzymes in the cellulosome, enhancing the cellulose breakdown efficiency. These findings align well with the molecular dynamics analyses discussed earlier.

Interparticle interactions, as long-range intermolecular interactions, can lead to an overestimation of the *R*_g_.^63^ This could account for the discrepancy observed between the experimental *R*_g_ values and those predicted by the *in silico* model. Moreover, while AlphaFold2 is a powerful tool for structural prediction, the dynamic nature and variety of possible conformations in very flexible domains can lead to an experimentally observed average *R*_g_ in the Guinier region that exceeds the *R*_g_ of the single structure predicted by the program. The *R*_g_ and the molecular weight calculated for *Pf*GH5 using the SAXS MoW 3.0 ^62^ web tool were 35.6 Å and 64.9 kDa, respectively. The molecular weight value determined is broadly consistent with that of a monomer (∼60 kDa) in solution. Furthermore, DLS results previously published also support the conclusion that *Pf*GH5 is a monomer in solution.^12^

Recent publications highlight the combined influence of steric repulsion and long-range intermolecular interactions (both attractive and repulsive) on the conformational and association equilibria, as well as the kinetics, of proteins and nucleic acids under crowded conditions.^64^ Macromolecular crowding provides a plausible explanation for the reduction in *R*_g_ as concentration increases.^64–66^ By limiting the available volume, crowding forces the protein into a more compact state, minimizing unfavorable interactions and thus decreasing the *R*_g_. Macromolecular crowding may be an important factor in the assembly and functional efficiency of cellulosomes.^65^ It may contribute to the formation of these large protein complexes by promoting protein–protein interactions within the crowded environment of the cellulosome. Future research employing *in vivo* studies and mimicking crowded conditions *in vitro* will be essential to fully elucidate these complex relationships.

As the experimental X-ray scattering curve indicated both high flexibility and interparticle interactions, we opted not to generate a low-resolution model. SAXS data for highly flexible proteins in solution represent an average of various conformations. A low-resolution model generated from such data may not capture all structural details, especially in highly mobile regions. These limitations make it difficult to reconstruct a single well-defined structure even at low resolution.

### 3.5 The Dockerin Module Enhances the GH5 Catalytic Domain Thermostability

To analyze the influence of the dockerin module on the GH5 catalytic domain, the isolated GH5 domain (*Pf*GH5_cat) was expressed and purified to homogeneity (Figure S11) using a previously described methodology.^12^ Circular dichroism (CD) spectroscopy was used to investigate the secondary structure and determine the melting temperature of the recombinant products in solution (Figure 7). The CD spectra of *Pf*GH5 and *Pf*GH5_cat exhibit negative ellipticities around 209 and 218 nm, characteristic of proteins containing α-helical secondary structures (Figure 7A).

**Figure 7:**
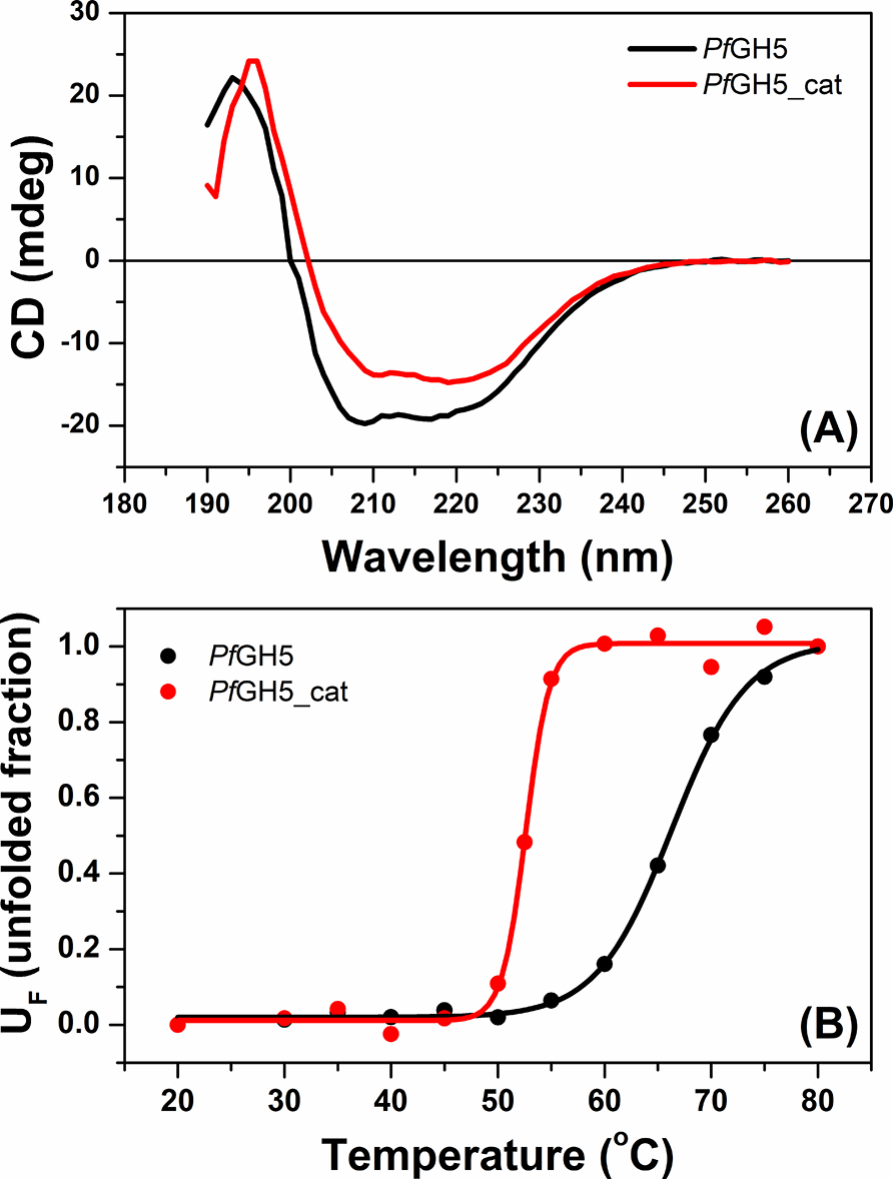
Thermal stability analysis. Analyses were performed under equimolar protein concentrations. (A) Circular dichroism spectra collected at pH 6 and 20 °C for *Pf*GH5 (black line) and *Pf*GH5_cat (red line). (B) The thermal denaturation of *Pf*GH5 and *Pf*GH5_cat was monitored at 220 nm as a function of temperature at pH 6.

The melting temperatures determined for *Pf*GH5 and *Pf*GH5_cat were 64 and 55 ± 1 °C, respectively (Figure 7B). As shown in Figure 7B, the melting temperature determined for *Pf*GH5_cat was significantly lower than that determined for the full-length protein, indicating that removing the dockerin module substantially reduces the thermostability of the isolated GH5 catalytic domain. Below 50 °C, the CD spectrum of *Pf*GH5_cat remains unchanged. However, upon exceeding this temperature, a gradual loss of regular secondary structures is observed. This coincides with *Pf*GH5_cat precipitation in a temperature-dependent manner, a behavior absent for the full-length protein. Few studies have investigated the effect of the dockerin domain on thermostability. To date, in contrast to our findings, Huang et al. reported a significant increase in melting temperature for xylanases lacking the C-terminal double dockerin module in Neocallimastix frontalis.^67^

### 3.6 The Carbohydrate-Binding Module Contributes to Improved Activity on Less Soluble Substrates

To assess the impact of the carbohydrate-binding module on GH5 domain activity, we compared the enzymatic activity of *Pf*GH5 and *Pf*GH5_cat against four different substrates as shown in Figure 8. Both *Pf*GH5 and *Pf*GH5_cat exhibited similar activity in the enzymatic hydrolysis of oat β-glucan, a linear polysaccharide composed of mixed-linkage (β-1,3 and β-1,4) glycosidic bonds, indicating a negligible influence of the carbohydrate-binding module. The presence of β-1,3 glycosidic bonds in oat β-glucan prevents overlap between the linear polymer chains, therefore contributing to the increase in the solubility and viscosity of this substrate when exposed to water.^68^ Furthermore, both *Pf*GH5 and *Pf*GH5_cat exhibited activity, though at lower rates, toward carboxymethylcellulose (CMC), β-mannan, and barley β-glucan. These findings revealed a broad range of enzymatic activities. This versatility likely stems from their ability to cleave β-1,4 glycosidic bonds within various substrates, a specificity further supported by cleavage pattern assays described in detail below.

**Figure 8:**
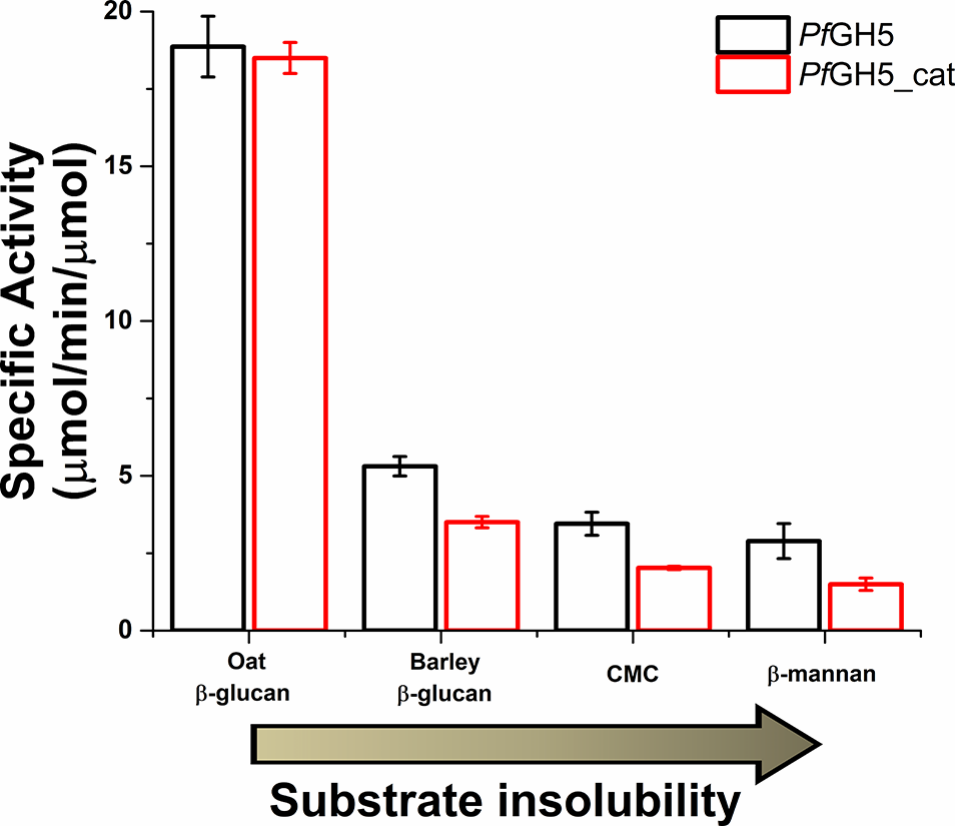
Substrate panel employing four different substrates (oat β-glucan, CMC, β-1,4-d-mannan, and barley β-glucan). Analyses were performed under equimolar protein concentrations. The black and red bars correspond to *Pf*GH5 and *Pf*GH5_cat, respectively. All assays were performed in triplicate. The substrates were ordered from most soluble to least soluble based on information obtained from their commercial manufacturer.

Substrates such as CMC and β-mannan are exclusively composed of β-1,4 glycosidic bonds, while oat β-glucan and barley β-glucan contain a mixture of β-1,3 and β-1,4 glycosidic bonds. Compared to the *Pf*GH5, *Pf*GH5_cat showed a decrease in enzymatic activity on less water-soluble substrates like β-mannan, CMC, and barley β-glucan (Figure 8). Collectively, our study revealed that the absence of the carbohydrate-binding module contributed to reducing the enzymatic activity of the catalytic domain toward less soluble substrates. This result highlights the role of the carbohydrate-binding module in facilitating interactions with substrates that have lower aqueous solubility.

### 3.7 The Effects of Dockerin Module on the Specific Activity Profiles

The effects of the dockerin module on the optimal temperatures of *Pf*GH5 and *Pf*GH5_cat are shown in Figure 9A.

**Figure 9:**
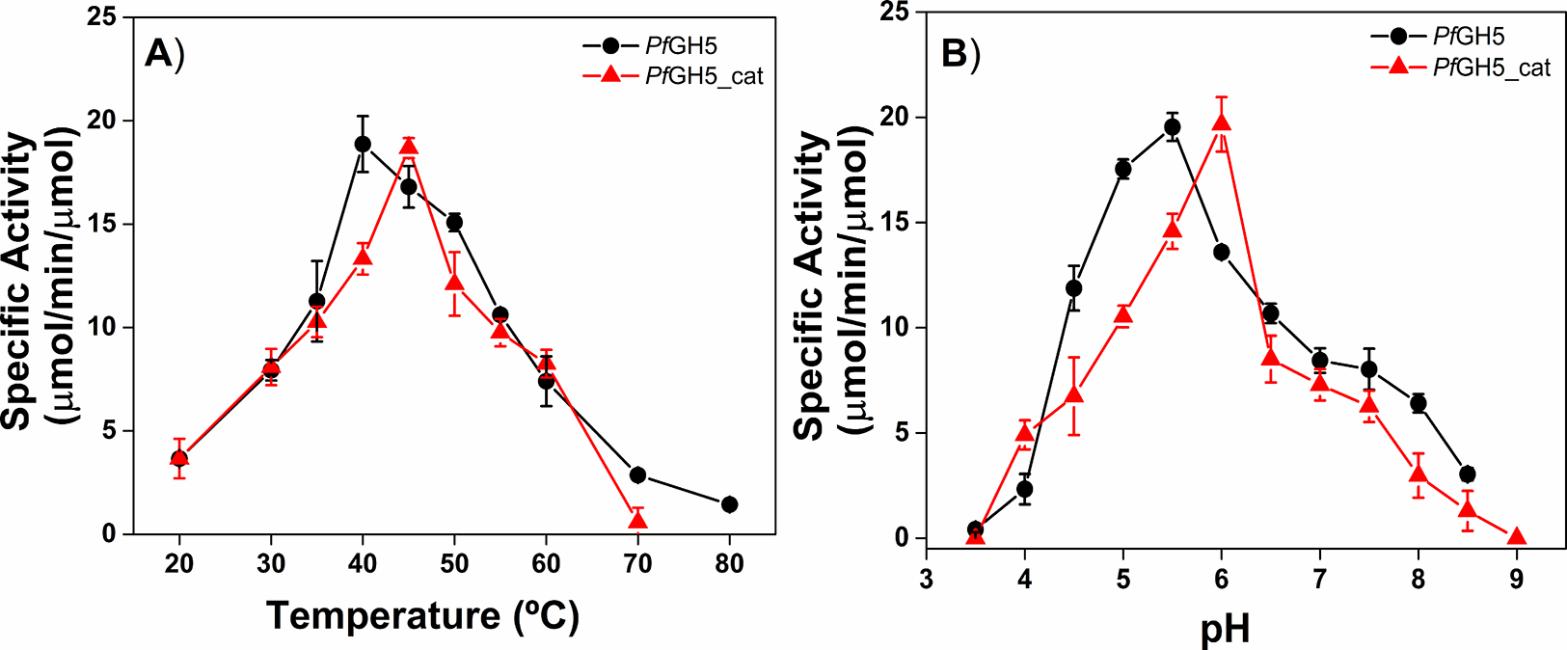
(A) Effect of temperature on the enzymatic activity of *Pf*GH5 (black circles) and *Pf*GH5_cat (red circles). (B) Effect of pH on the enzymatic activity of *Pf*GH5 (black circles) and *Pf*GH5_cat (red circles). Analyses were performed under equimolar protein concentrations. The solid line is just a guide for the eyes.

*Pf*GH5 and *Pf*GH5_cat exhibited similar specific activity profiles with optimal temperatures at 40 and 45 °C, respectively. Therefore, removing the dockerin module resulted in a slight increase of 5 °C in the optimal temperature for *Pf*GH5_cat. Studies have shown varying effects of dockerin modules on enzyme activity. Removing the double dockerin modules from N. frontalis Xyn11A and Xyn11B resulted in an increase in specific activity and a 5 °C elevation in optimal temperatures.^67^ This latter observation is consistent with our findings. In contrast, removing the C-terminal dockerin module from *Piromyces* mannanase had no impact on its activity.^54^ Figure 9B shows the effects of the dockerin module on the optimal pH values. *Pf*GH5 and *Pf*GH5_cat showed similar specific activity profiles with optimal pH values of 5.5 and 6.0, respectively. Therefore, removing the dockerin module caused a slight increase in the optimal pH for *Pf*GH5_cat. In contrast, optimal pH remained unchanged when the double dockerin modules from N. frontalis Xyn11A and Xyn11B were removed.^67^ A recent study reported the addition of fungal dockerin modules to bacterial enzymes for the generation of chimera enzymes.^24^ This study investigated the effect of adding a fungal dockerin module, present in the genome of the fungus P. finnis, onto a GH5 catalytic domain from the hyperthermophilic bacterium Thermotoga maritima named Cel5A. The results showed no influence on the specific activity, optimal temperature, or optimal pH when comparing the chimera enzyme to the GH5 domain isolated. Similarly, a previous study found that the enzyme kinetics of CelD from P. finnis remained unaffected by the presence or absence of its native C-terminal dockerin modules or the addition of a non-native N-terminal dockerin module.^49^

### 3.8 The Effects of Dockerin Module on the Hydrolytic Degradation Patterns

The hydrolytic cleavage patterns of *Pf*GH5 and *Pf*GH5_cat on cellohexaose (C6) were monitored by using ion chromatography. This analysis aimed to investigate whether removing the dockerin module caused any changes in the enzyme’s hydrolytic mechanism, as depicted in Figure 10 and Figure S12.

**Figure 10:**
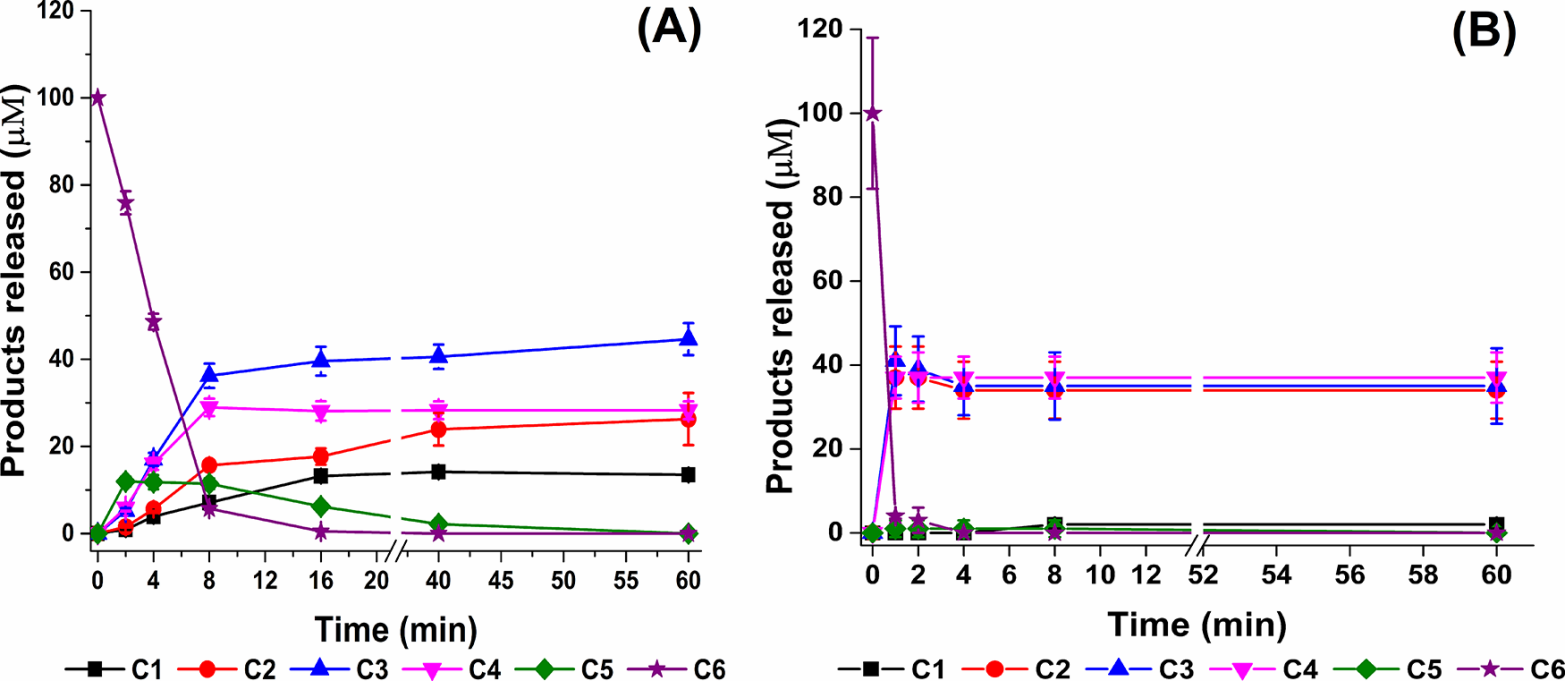
Hydrolytic degradation profile for cellohexaose (C6). Analyses were performed under equimolar protein concentrations. (A) *Pf*GH5. (B) *Pf*GH5_cat. Analyses performed by ion exchange chromatography (HPAEC) coupled with pulsed amperometry (PAD) in the ICS-6000 Dionex. The black, red, blue, magenta, green, and purple lines correspond to glucose (C1), cellobiose (C2), cellotriose (C3), cellotetraose (C4), cellopentaose (C5), and cellohexaose (C6), respectively.

*Pf*GH5_cat (Figure 10B) rapidly released equal amounts of cellobiose (C2), cellotriose (C3), and cellotetrose (C4) within the first minute of the reaction. In contrast, *Pf*GH5 exhibited a slightly different profile (Figure 10A), requiring more time (approximately 8 min) to release different amounts of C2, C3, and C4. These data suggest that the absence of the dockerin module potentializes endoglucanase catalytic domain random attack on cello-oligosaccharides, increasing its initial catalytic efficiency.

## 4 Conclusions

This study reports the first structural characterization of a modular anaerobic fungal cellulase containing a dockerin module. Our analyses revealed that the endoglucanase from P. finnis has a nonglobular conformation, exhibiting high flexibility in solution due to two main collective motions: bending and twisting. The role of the dockerin module goes beyond simple structural attachment to the cellulosome. It enhances the thermostability of the catalytic domain, a property that could be exploited to improve the enzyme performance under industrial conditions. Furthermore, the removal of the dockerin module resulted in a slight increase in the optimal temperature and pH values of the isolated catalytic domain and favored the random attack on soluble cello-oligosaccharides. The lack of the carbohydrate-binding module hampered activity toward less soluble substrates. The high flexibility observed for the anaerobic full-length *Pf*GH5 in this study could allow it to not only reach cellulose chains of difficult access within the substrate but also improve its synergic effect with other enzymes within the cellulosomal supramolecular structure. By shedding light on the role of the dockerin module in anaerobic fungal cellulases, this study provides novel and valuable insights that are helpful in applications involving lignocellulosic biomass conversion into biofuels and bioproducts.

## Supplementary Material



## Notes

Original sequence prospected in accession: PRJNA 291757 (Table S1); secondary structure content comparison of *Pf*GH5 domains (Table S2); Predicted Local Distance Difference Test (pLDDT) (Figure S1); Ramachandran plot (Figure S2); predicted aligned error (PAE) from the selected AlphaFold2 model (Figure S3); contact map for the AlfaFold2 model and minimized and equilibrated structure (Figure S4); DSSP map for the AlphaFold2 model and Classical Molecular Dynamics (Figure S5); average contact map for Classical Molecular Dynamics (Figure S6); radius of gyration (*R*_g_) over time for Classical Molecular Dynamics (MD) (Figure S7); histogram of RMSD-VMOD and MDeNM (Figure S8); root mean square deviation (RMSD) of the protein structure over simulation time (Figure S9); fluctuation of α-carbon by residues for the first 20 modes (Figure S10); size-exclusion chromatography (SEC) and SDS-PAGE (Figure S11); and enzymatic reactions containing cellohexaose (C6) (Figure S12) (PDF)
