# Supplementary material for: Dissection of an Anaerobic Fungal Cellulosomal Endoglucanase: Impact of the Dockerin Module on Activity, Thermostability, and Flexibility
Source: ACS Omega. 2025 May 14;10(20):20474–86. doi: 10.1021/acsomega.5c00685 (PMC12120621; doi:10.1021/acsomega.5c00685)

Supporting Information

Dissection of an anaerobic fungal cellulosomal endoglucanase: impact of the dockerin module on activity, thermostability, and flexibility

Viviane Brito Andrade[a], Robson Tramontina[b], Dnane Vieira Almeida[a], Geizecler Tomazetto[b], Viviam M da Silva[d], Frank Gabel[d], Yolanda M B Marcello[e], Ana Ligia Scott[e], Fabio Marcio Squina[f], Wanius Garcia[a,*]

[a] Universidade Federal do ABC (UFABC), Centro de Ciências Naturais e Humanas (CCNH), Avenida dos Estados 5001, Bairro Bangu, CEP 09280-560, Santo André, SP, Brazil.

[b] Universidade Estadual de Campinas (UNICAMP), Department of Biochemistry and Tissue Biology, Institute of Biology, Rua Monteiro Lobato 255, CEP 13083-970, Campinas, SP, Brazil.

[c] University of Pittsburgh, School of Medicine, Department of Pediatrics, 4401 Penn Avenue, Pittsburgh, PA, United States.

[d] Institut de Biologie Structurale (IBS), CEA, CNRS, UGA, 71 Avenue des Martyrs, CS 10090, 38000 Grenoble, France.

[e] Universidade Federal do ABC (UFABC), Centro de Matemática, Computação e Cognição (CMCC), Avenida dos Estados 5001, Bairro Bangu, CEP 09280-560, Santo André, SP, Brazil.

[f] Universidade de Sorocaba (UNISO), Laboratório de Ciências Moleculares, Rodovia Raposo Tavares, km 92,5, CEP 18023-000, Sorocaba, SP, Brazil.

[*] Corresponding author at: Universidade Federal do ABC (UFABC), Centro de Ciências Naturais e Humanas (CCNH), Avenida dos Estados 5001, Bairro Bangu, CEP 09280-560, Santo André, SP, Brazil, orcid.org/0000-0003-3712-3488, E-mail: wanius.garcia@ufabc.edu.br.

Index

**Table S1.** Original sequence prospected in accession: PRJNA 291757.	**03**
**Table S2.** Secondary structure contents comparison of *Pf*GH5 domains.	**04**
**Figure S1.** Predicted Local Distance Difference test (pLDDT)	**05**
**Figure S2.** Ramachandran plot.	**06**
**Figure S3.** Predicted Aligned Error (PAE) from the selected AlphaFold2 model.	**07**
**Figure S4.** Contact map for AlfaFold2 model and minimized and equilibrated structure	**08**
**Figure S5.** DSSP map for AlphaFold2 model and Classical Molecular Dynamics.	**09**
**Figure S6.** Average contact map for Classical Molecular Dynamics.	**10**
**Figure S7.** Radius of gyration (Rg) over time for Classical Molecular Dynamics (MD).	**11**
**Figure S8.** Histogram of RMSD - VMOD and MDeNM	**12**
**Figure S9.** Root Mean Square Deviation (RMSD) of the protein structure over simulation time.	**13**
**Figure S10.** Fluctuation of α-carbon by residues for the first twenty modes.	**14**
**Figure S11.** Size-exclusion chromatography (SEC) and SDS-PAGE.	**15**
**Figure S12.** Enzymatic reactions containing cellohexaose (C6).	**16**

Table S1. Original sequence prospected in accession: PRJNA 291757.

>COMP11848_C2_SEQ3 LEN=2477 PATH=[5708:0-696 116:697-1901 1320:1902-2026 1445:2027-2476]

```
AGCTGGTGATGCTTTTGTTACGGTTACTGAACAAGATACGGCTCTTTGCAATTCCAAATTTGAAAATA
CTTTTAATTTTTATAATTAAAGTAATTTTGGAATTTATATTAAATCGAAATTTATAAAAATATATACAA
TATTCATTAAACCATTAAAAAGAAGAAATGAAAAGAATATATATTATTATATTATGATTATGCTATTT
CATTATTAATTCATATTCATGATTAATAAATTCCAATCTTTAGAAATTAGATAAAATAGATGAATATA
AGTAAAAATATGAAAGTAAAATAAGATATAAAATAATAAAAATAGAAATAATTATGATATATTATAT
ATAAAACAATTAGTTTCTATTTAATTTTTTAATGAGCTTATTATTAATATAGATTTAATTTTGAATGTTT
ACTATAGATAAAATACATTAAAAAAAAATGGAAAAATATTAAAAAATAGGTTTTGATTTAGTTTTATA
AAAAAAAAAAAGTATAGAAAAAATTATAAAAATAGTAACAATAGTATACTCCAATAAATAAAACTAA
AACTAAACCTTTGATAAATATAAAAATATAAAAAAAATATTGTCTGGAACAATATTTGGGATAAATTA
TTTGAAATGAAATTCTCAAAAGCTTTATTTTTATCATCTTTGGCTTATGCCTCAGCCAAAAAACTTCAA
TATTTGGGTGTGAATGAATCCAGTGGTGAATTTGGTGAAGGTAATTTACCAGGTGTTTACAATAAACA
TTATATTTATCCAGATGTTAAAGCCATCGAAACTACTATCGAACAAGGTATGAACGCTTTCCGTATTTG
TAACAGATGGGAACGTTTACAGCATGAATTATTTGGTGAATTTAATGAATTCGATATCACTGAATTTA
AAAAGGTTGTTGATGCTACTACTGCTAAGGGTGCCATTGCTATTATTGATCCACATAACTATGCTCGTT
ACAACAATAAACTTATTGGTTCTGAAGATGTTCCAATTGAAGCTTTCGTTGACTTCTGGACTAGACTTG
CTGAAATTTTCAAAGATAATGAAAATGTTTGGTTCGGTTTAGTTAATGAACCACACGATATGGAAACT
GATGATTGGTTCAAGGCAGCTCGTGCTGCTGTTGATGGTATTCGTTCCACTGGTGCTAAGAACAACAT
TTTAATTCCAGGTAACGGTTGGACTGGAGCTTGGAGTTGGGGTAAGGAAGCTTGGTATGGTGAAGCTA
ATGCTGATGTTGCCTTAAGATATTTCTCATCTGAAGATGAAAATATTCTTTTTGAAGTTCACCAATACT
TTGATAAAGATTACTCTGGTACAGGTGATCAATGTGTTCAACGCCCATGTCAAAATCTCTTTAAAGAA
TTTGTTGAATGGCTTAAGACCAACAATTTAAAGGGATGGATTGGAGAAATTGGTTCATACCTTACTGA
TGAATGCCGTGAATGTGTTCAAGAATCCATTGAATATCTCCAAGAAAACAATGAATATGTTCTTGGTA
CTTTATGGTGGGCAGCAGGTCCATGGTGGGGACATAATGCTATGTCTATTGAACCAAATGCCGAAAAA
GCATTCCCAGGACAAATGGCATGGTTAAAGCCATATTTACCAGGACCATCCGAATTAACTGAAGTTCC
AACCTTCATTAACAAGAAGGTTTACTGTGAAGGATGTGTTGTCACAGGTACTGGTGGTGACGGTTCTT
TATGGGGATGGGAAAATGAAAAGTCCTGTGAAATTGATATAGAAACTTGTGGATACAATTCAGGAAC
CTCAATTGACAATGGAAATACTTCAACTGAAAAAACATACTGTAAGGGATGTGTTGTTACAGGTACTG
GTGGTGATGGTTCCTTATGGGGATGGGAAGATGAAAAGTCATGTGAAATTGATGTTAAAAAATGTGA
AACTGTTGATGAATGTTGGTCTGAAGCTTTAGGTTACAAATGCTGTTCTCACTGCGTTGCTATATTAGA
AGATCAAGATGGTGCATGGGGTTCTGAAAATGGTGAATGGTGTGGTATTCCAGATACTTGTCCAACCT
CTACTAAGAATAAGCGTAGTGATGGTTTTACCATTCTTCCAATTTGCCACAACCCATTACCAACTTGTG
TTGCTTTATATGATCAATGTGGTGGTGATGGCTACTTTGGTCCAACTGAATGTTGTAAGGGAAAATGT
ATTTCTCAAGGACCATACTATTCCCAATGTGCTGAAGAAGAATAAATCATATTAGAAAAGTAAAAGA
ATAAAAAAAATCCAAATAACAAGTTTATAAATTTTATTAAAAGAATATATACATTGCTTGAAAATTCA
AAAGCAAAAAAATAAGTACTAATAAAAGAAATAATAGTAATGGAATAATTTTAAAAAAAAAAAAATGTT
TTCTTAACTTTGTAATATATGAAAAACAAAAATAAAAATAAATTAATTTTAAAAAAAAAAAAAAAAA
ATAAGAAGAAAAAAAAAAAAAAAAAA
```

> VIRT-3703244:5'3' Frame 2, start_pos=206

```
MKFSKALFLSSLAYASAKKLQYLGVNESSGEFGEGNLPGVYNKHYIYPDVKAIETTIEQGMNAFRICNRWE
RLQHELFGEFNEFDITEFKKVVDATTAKGAIAIIDPHNYARYNNKLIGSEDVPIEAFVDFWTRLAEIFKDNE
NVWFGLVNEPHDMETDDWFKAARAAVDGIRSTGAKNNILIPGNGWTGAWSWGKEAWYGEANADVALR
YFSSEDENILFEVHQYFDKDYSGTGDQCVQRPCQNLFKEFVEWLKTNNLKGWIGEIGSYLTDECRECVQES
IEYLQENNEYVLGTLWWAAGPWWGHNAMSIEPNAEKAFPGQMAWLKPYLPGPSELTEVPTFINKKVYCE
GCVVTGTGGDGSLWGWENEKSCEIDIETCGYNSGTSIDNGNTSTEKTYCKGCVVTGTGGDGSLWGWEDE
KSCEIDVKKCETVDECWSEALGYKCCSHCVAILEDQDGAWGSENGEWCGIPDTCPTSTKNKRSDGFTILPI
CHNPLPTCVALYDQCGGDGYFGPTECCKGKCISQGPYYSQCAEEE
```

The underlined amino acid residues refer to the signal peptide.

Table S2. Secondary structure contents comparison of *Pf*GH5 domains.

Domain	Method	α-helix (%)	β-strand (%)	Coil (%)
GH5 (PDB: 8GHX) *Piromyces finnis*	X-ray crystallography	38.38	11.48	50.14
CBM1 (PDB: 7YHI) *Trichoderma reesei*	Nuclear Magnetic Resonance	0.00	25.00	75.00
CBM10 (PDB: 2J4N) *Piromyces equi*	Nuclear Magnetic Resonance	4.17	24.07	71.76
GH5	In silico model: minimized and equilibrated structure	34.08	18.15	47.77
CBM1		0	38.89	61.11
CBM10		13.51%	32.43%	54.05%
GH5	In silico model: Classical Molecular Dynamics (100 ns)	34.04	16.37	49.60
CBM1		0.02	34.30	65.69
CBM10		12.44	34.23	53.33
GH5	In silico model: MDeNM (20 x 10 ps)	34.02	17.33	48.65
CBM1		0.02	32.60	67.38
CBM10		8.88	33.34	57.78

Figure S1. Predicted Local Distance Difference test (pLDDT), which was on average 97 and 60 in the most structure. Values between 60 and 96 indicate a good accuracy, where the prediction of the main chain of the protein is reliable. Some residues on the links regions presents a lower value on the graphs that is expected because they are in small very flexible. We selected the best model and after AlphaFold2, we performed a Minimization and a long Molecular Dynamics to improve the quality of the model in these regions.

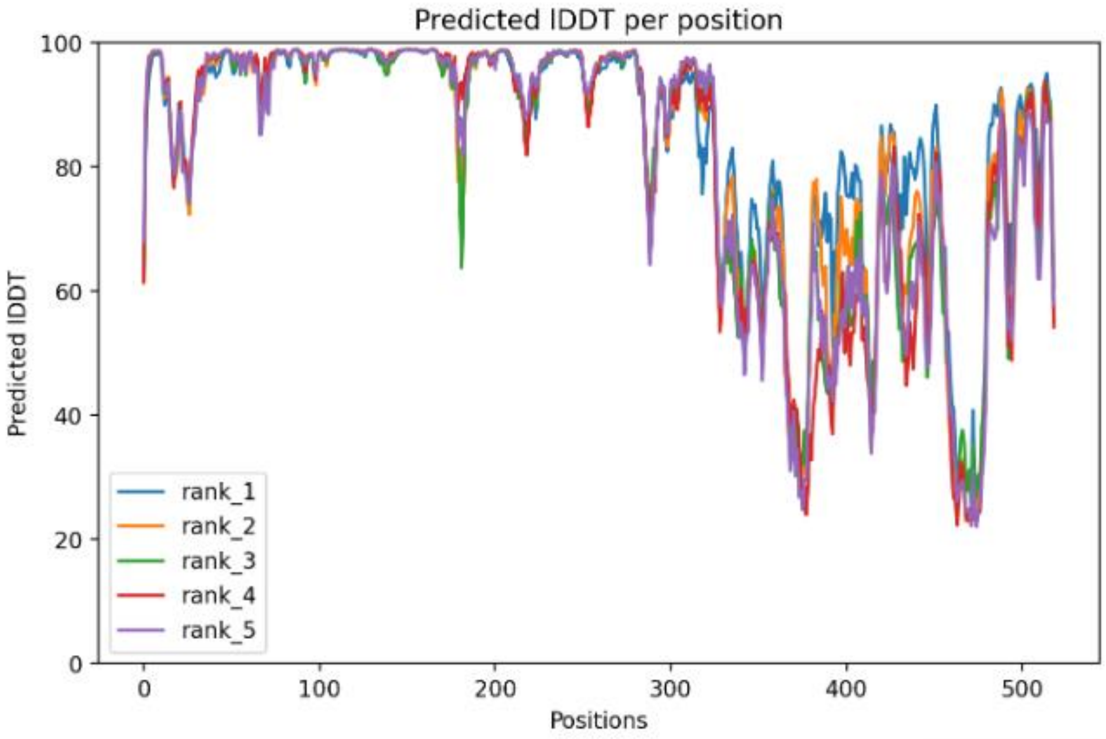

Figure S2. Ramachandran plot for the *Pf*GH5 final model generated by AlphaFold2 program and Molecular Dynamics Simulation.

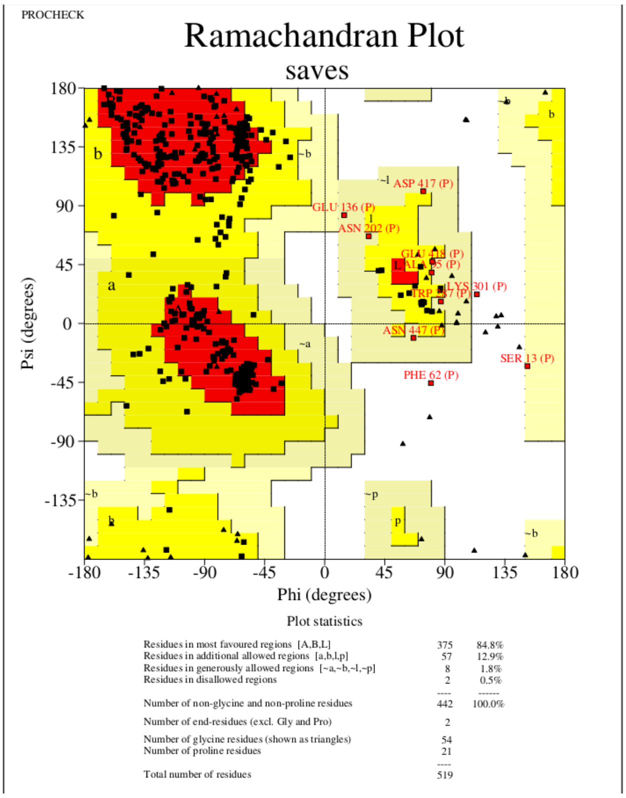

Figure S3. Predicted Aligned Error (PAE) from the selected AlphaFold2 model. The color scale bar on the right side indicates the PAE values, where lower values correspond to a higher-quality model.

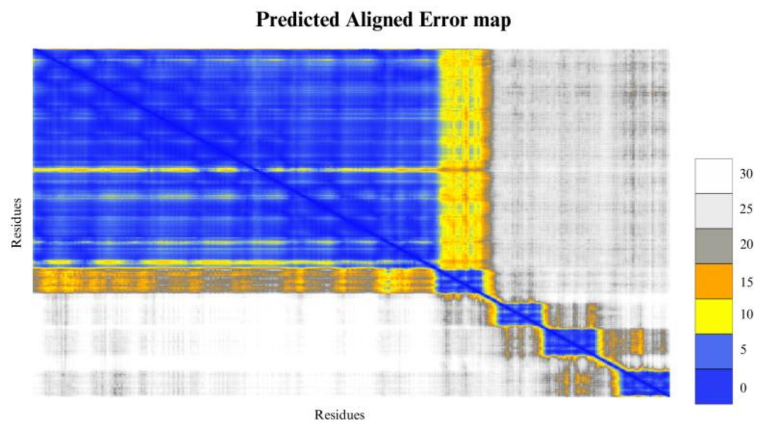

Figure S4. Contact map for A) AlfaFold2 model, and B) minimized and equilibrated structure.

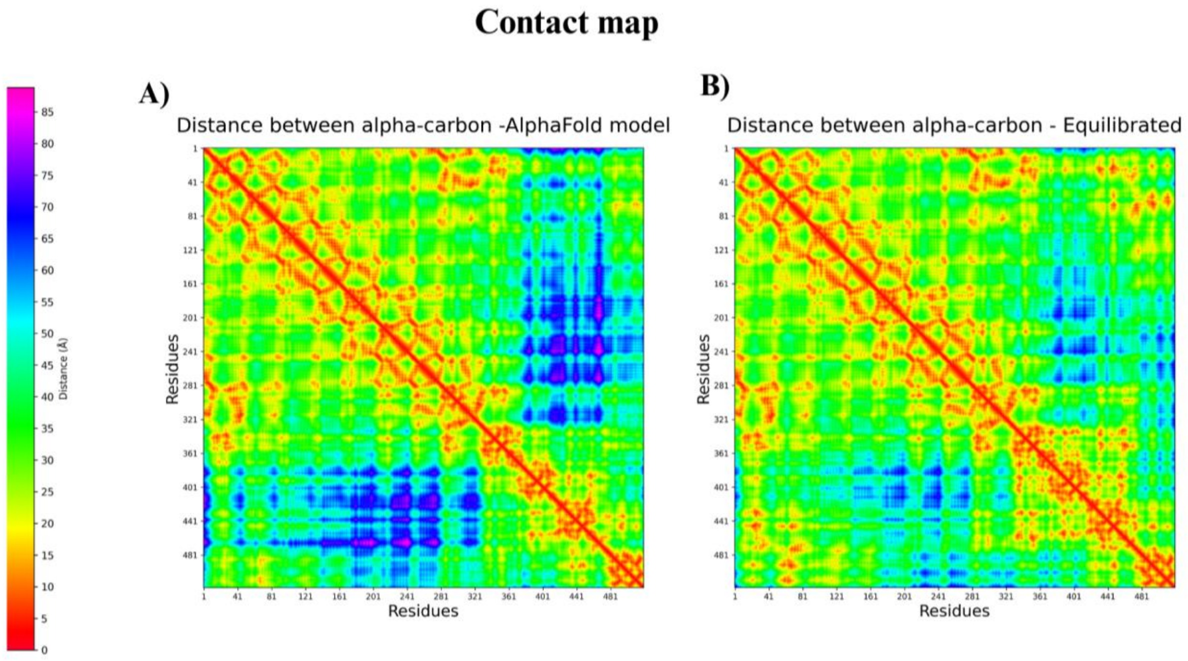

Figure S5. DSSP map for: A) AlphaFold2 model, and B) Classical Molecular Dynamics. This plot uses the following notation: H for α-helix, B for isolated β-bridge, E for β-strand, G for 3-10 helix, I for π-helix, T for turn, S for bend, and - for coil.

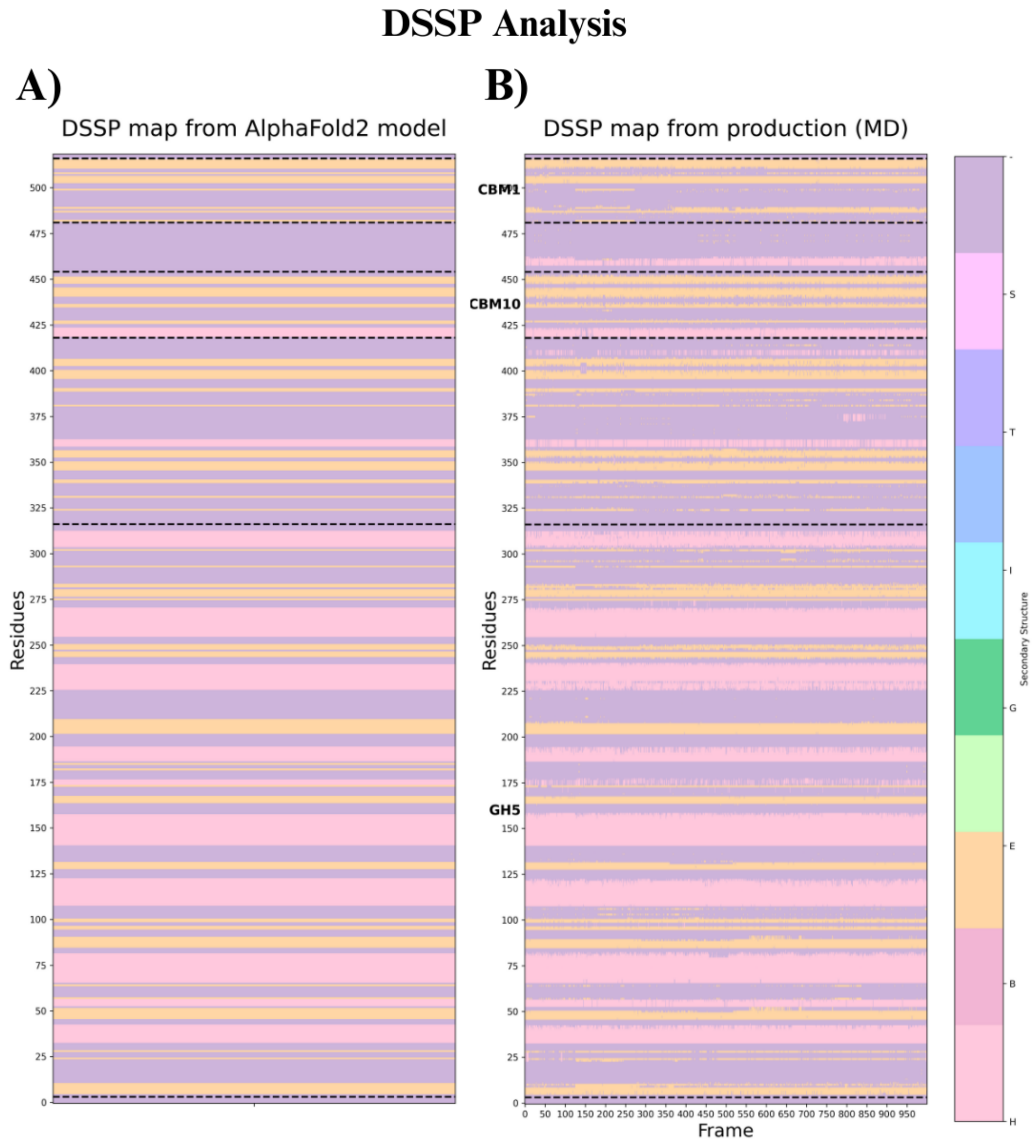

Figure S6. Average contact map for Classical Molecular Dynamics.

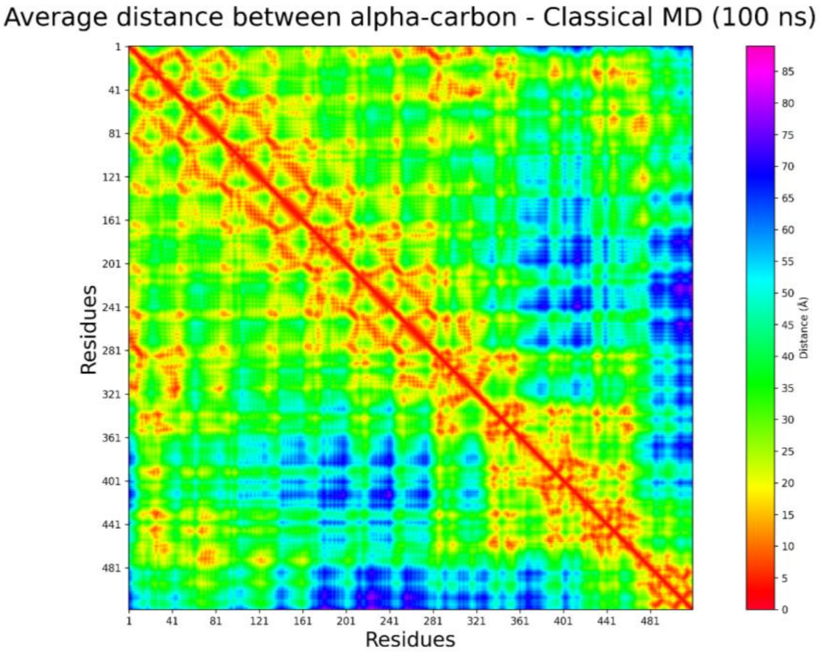

Figure S7. Radius of gyration (Rg) over time for Classical Molecular Dynamics (MD).

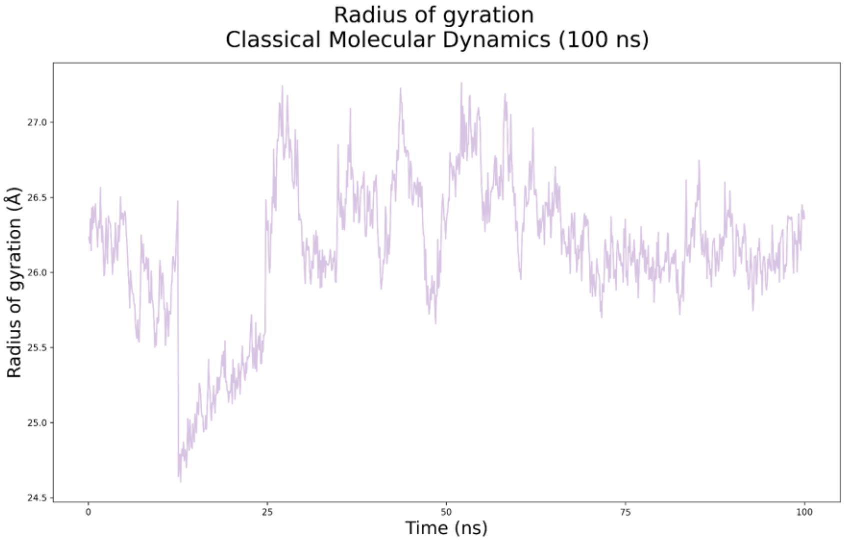

Figure S8. A) Histogram of RMSD for the conformations obtained by VMOD. These values were calculated with respect to the initial model. **B)** Histogram of RMSD calculated between the conformations generated by MDeNM and the initial model.

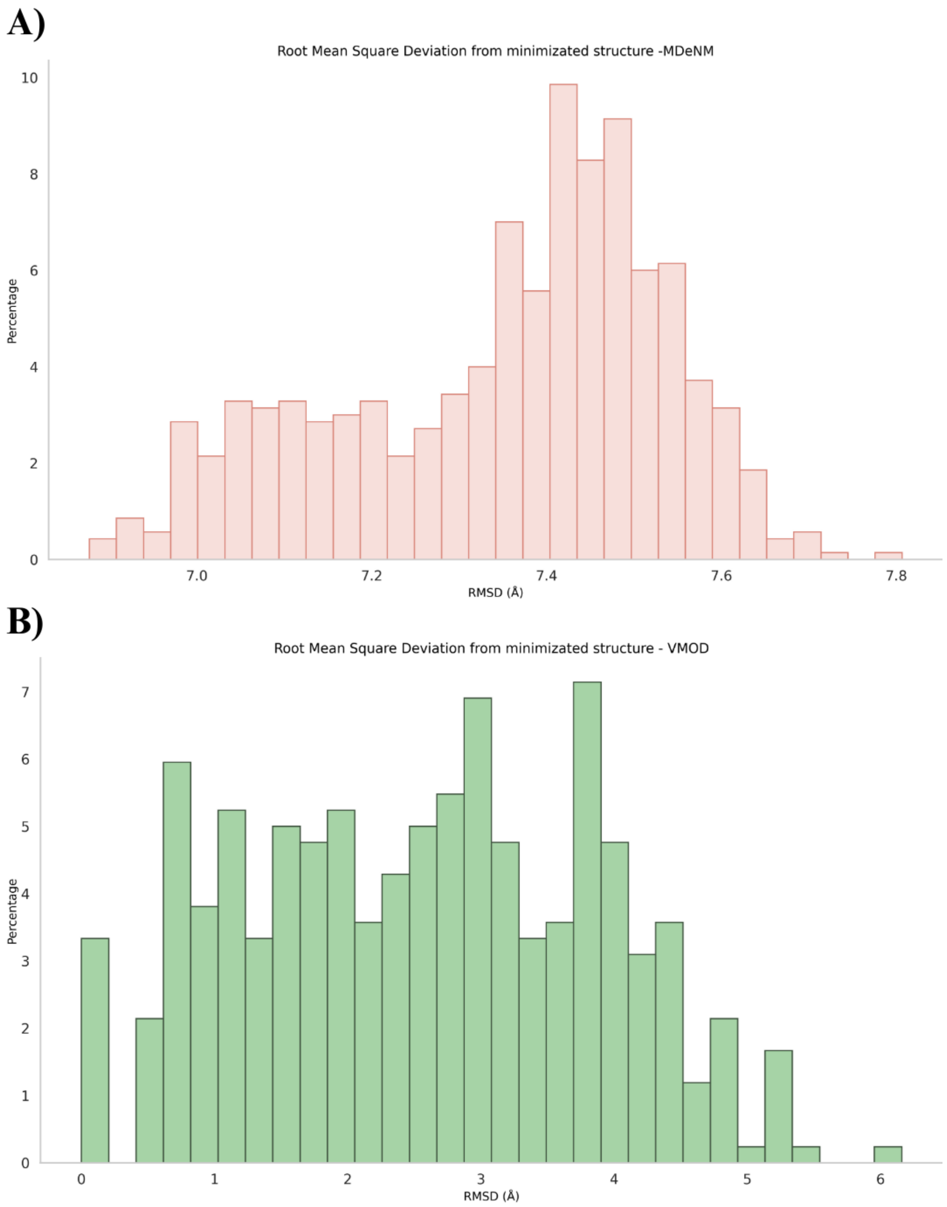

Figure S9. Root Mean Square Deviation (RMSD) of the protein structure over simulation time.

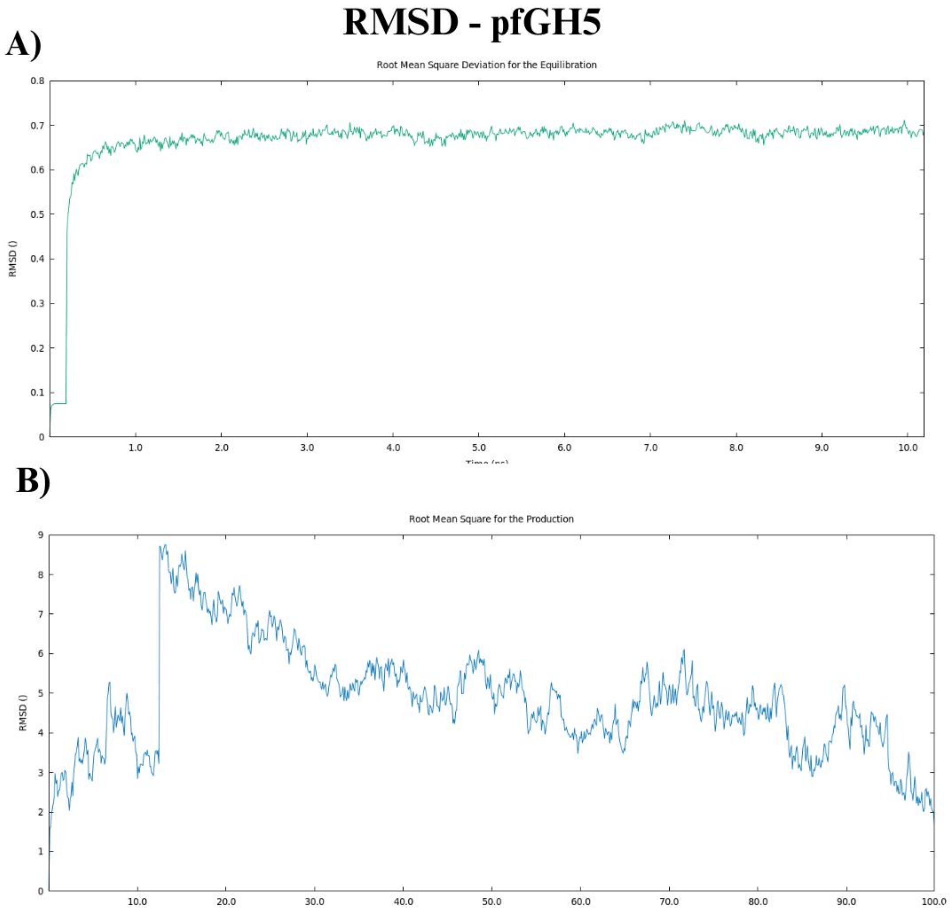

Figure S10. Fluctuation of α-carbon (C_α) by residues for the first twenty modes.

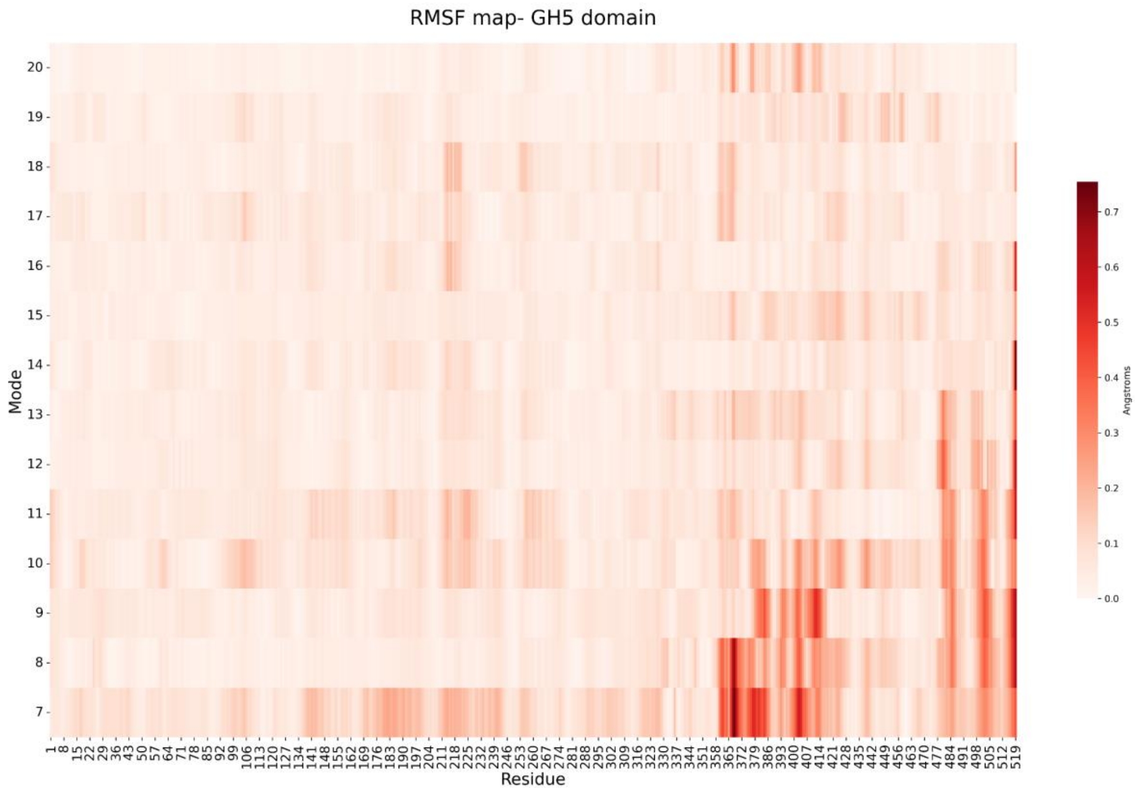

Figure S11. Size-exclusion chromatography (SEC). **A**) SEC of *Pf*GH5. Inset: Lane 1, molecular marker. Lanes 2 and 3, SEC fractions (elution volumes are shown). **B**) SEC of *Pf*GH5_cat. Inset: Lane 1, molecular marker. Lanes 2 and 3, SEC fractions (elution volumes are shown).

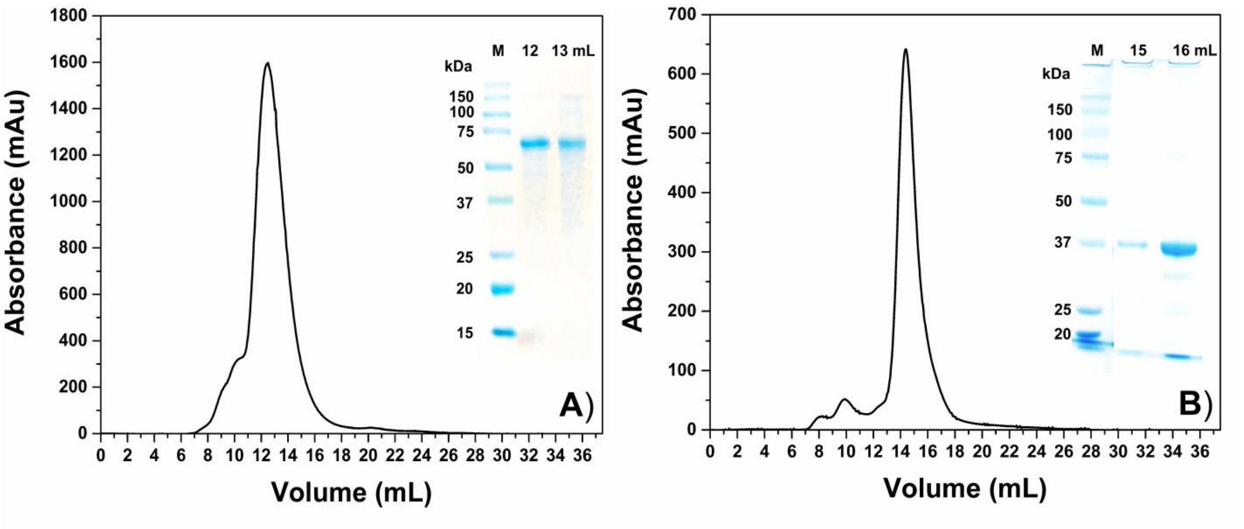

Figure S12. Enzymatic reactions containing cellohexaose. **A)** *Pf*GH5. **B)** *Pf*GH5_cat. The reactions were monitored by ion exchange chromatography (HPAEC) coupled with Pulsed Amperometric (PAD) in the equipment ICS-6000 Dionex (Thermo Scientific). The data were analyzed using the Chromeleon Chromatography Data System. The y-axis is given in nano Coulomb (nC).

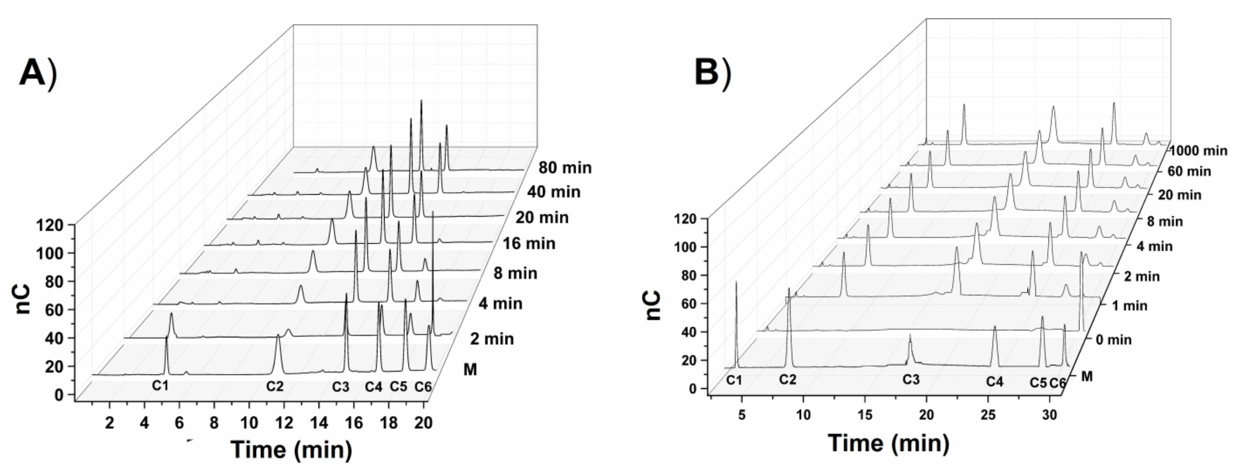

Supplement: Supplementary file 1 [file ao5c00685_si_001.pdf]
